# Seeing Bacterial Persistence Through Different Experimental Lenses: How Methodology Shapes Biological Interpretation

**DOI:** 10.3390/pathogens15090964

**Published:** 2026-09-09

**Authors:** Karolina Stojowska-Swędrzyńska, Dorota Kuczyńska-Wiśnik, Ewa Laskowska

**Affiliations:** Department of General and Medical Biochemistry, Faculty of Biology, University of Gdańsk, Wita Stwosza 59, 80-308 Gdańsk, Poland; dorota.kuczynska-wisnik@ug.edu.pl (D.K.-W.); ewa.laskowska@ug.edu.pl (E.L.)

**Keywords:** antibiotic treatment, CFU, cell sorting, FACS, microfluidics, fluorescence microscopy, persister cells, population-based analysis, population enrichment, single-cell analysis

## Abstract

Bacterial persister cells are a rare subpopulation that survives antibiotic treatment without acquiring heritable resistance. Despite decades of research, concepts of bacterial persistence have evolved alongside experimental methods, yet the mechanisms underlying persistence remain debated, and different approaches often lead to seemingly conflicting conclusions. In this Review, we argue that these discrepancies arise not only from the heterogeneity of persister cells but also from differences in experimental methodology and biological context. We present persistence as a dynamic continuum extending from the physiological state preceding antibiotic exposure, through survival during treatment, recovery after antibiotic removal, and clonal expansion. We examine how experimental approaches—including CFU assays, population-based analyses, fluorescence microscopy, microfluidics, enrichment strategies, and cell sorting—interrogate different stages of this continuum, thereby addressing distinct biological questions rather than providing equivalent views of persistence. We further discuss how experimental context shapes the populations available for analysis and influences the interpretation of persistence phenotypes. Seemingly conflicting observations often arise from different experimental frameworks, each revealing a distinct aspect of bacterial persistence. Combining approaches that interrogate different stages of the continuum can strengthen persister identification and mechanistic interpretation. Recognizing these complementary perspectives is essential for accurately interpreting persistence and integrating findings across studies.

## 1. Introduction

Bacterial persistence has emerged as one of the central concepts in antimicrobial research because of its contribution to treatment failure, recurrent infections, and the evolution of antibiotic resistance. Although persister cells have been investigated for decades, one fundamental question remains surprisingly difficult to answer: what exactly is the bacterial cell that we call a persister?

At first glance, the answer appears straightforward. Persisters are cells that survive exposure to bactericidal antibiotics without acquiring heritable antibiotic resistance and subsequently resume growth once the antibiotic is removed [1]. However, this seemingly simple definition conceals a fundamental experimental paradox: a persister cannot be identified before the experiment begins, but at its end, only after the cell has survived antibiotic treatment and resumed growth. Persisters lack a universal genetic determinant, molecular marker, or physiological characteristic and are therefore recognized only retrospectively by their fate [1,2,3].

If persisters are defined by survival and regrowth but are studied before either event occurs, to what extent do experimental methods determine which bacterial cells can be studied and ultimately classified as persisters? Could apparently conflicting models of persistence arise, at least in part, from differences in experimental methodology rather than from fundamental biological disagreement?

In this Review, we examine persistence from a methodological perspective by asking how experimental methods and biological context have shaped our understanding of bacterial persistence. We argue that experimental approaches do more than improve analytical resolution. Together with the biological context in which persistence is investigated, they define which bacterial cells become accessible for analysis, which stages of the persistence continuum can be observed, and consequently, which biological conclusions can be legitimately drawn.

Literature relevant to the scope of this Review was identified through searches of PubMed, Web of Science, and Google Scholar, complemented by reference-based searches of relevant publications. No predefined publication date limits were applied, allowing both landmark studies and recent methodological developments to be considered. Searches combined general terms such as “bacterial persistence” and “persister cells” with method- or question-specific terms relevant to the topics discussed in each section, including “CFU assay”, “single-cell microscopy”, “microfluidics”, “enrichment”, and “cell sorting”.

As this is a narrative Review, literature selection was guided by conceptual and methodological relevance. The Review focuses on experimental studies that introduced major methodological advances or fundamentally changed the interpretation of persister biology. Wherever possible, emphasis is placed on recent developments, while landmark studies are included as needed to provide historical context for methodological innovations and conceptual advances.

Accordingly, the following sections are organized around the experimental questions addressed by each methodological approach and the biological insights these approaches make accessible.

## 2. Persistence as a Biological Continuum: A Framework for Understanding Experimental Approaches

**Figure 1:**
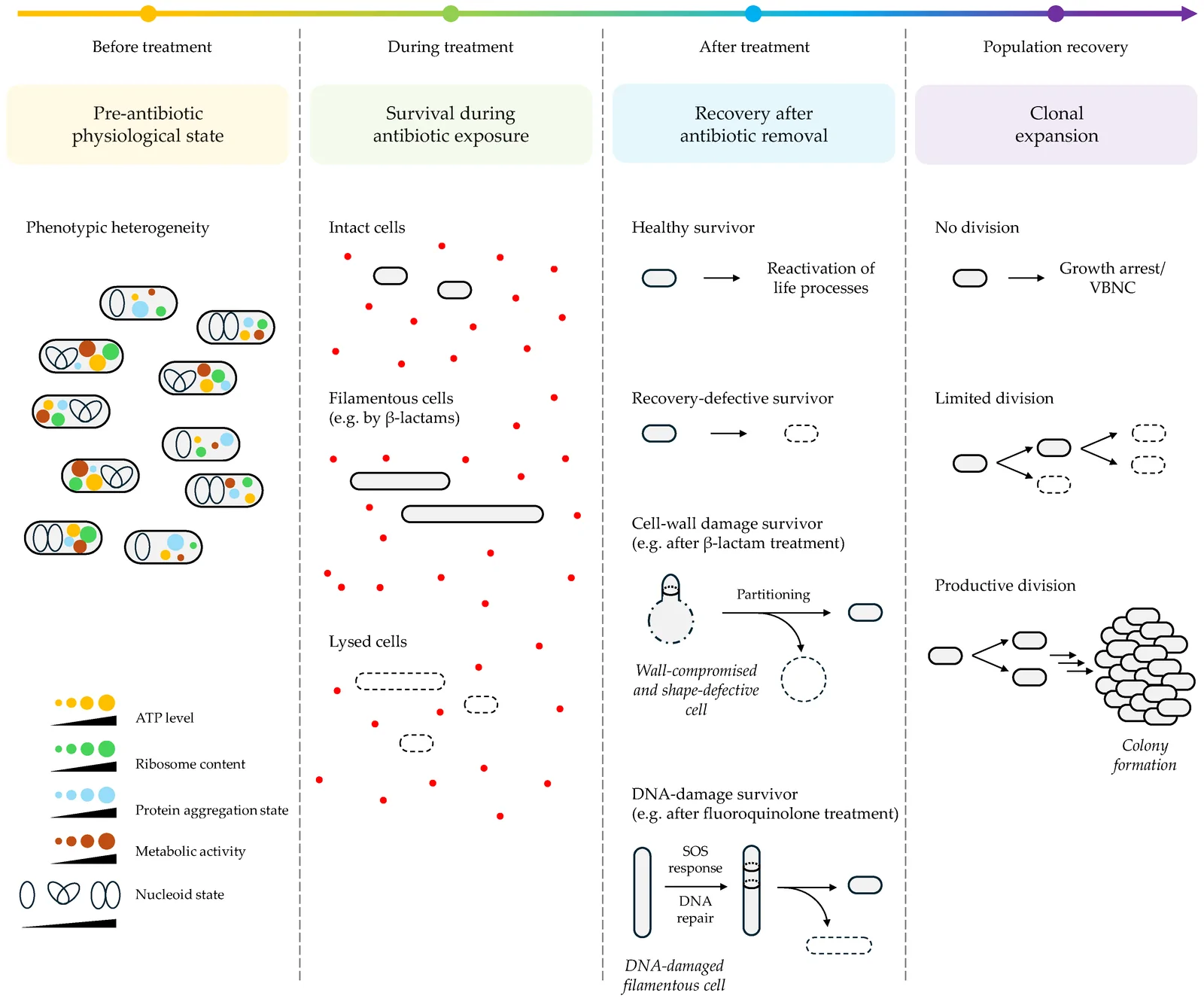
Persistence as a biological continuum comprising four conceptually distinguishable stages extending from the physiological state preceding antibiotic exposure to clonal expansion. Each stage constitutes a biological selection step in which individual cells follow different trajectories; only a subset successfully progresses through the continuum to ultimately form a colony, whereas others undergo lysis, irreversible damage, growth arrest, or enter the VBNC state. In the pre-antibiotic physiological stage, colored dots represent selected physiological parameters, whereas dot size indicates their relative level or abundance. During antibiotic exposure, red dots represent antibiotic molecules. The recovery and clonal expansion stages illustrate representative cellular trajectories and proliferation outcomes following antibiotic treatment and are not intended to represent all possible recovery pathways. The morphological response to antibiotic treatment depends on the antibiotic’s mechanism of action. Consequently, different antibiotics emphasize distinct stages and determinants of the persistence continuum [3,4,5,6,7,8,9,10,11,12,13].

The paradox introduced in the previous section becomes easier to understand when persistence is viewed not as a single measurable phenotype, but as a multistage biological process. To provide a common framework for comparing experimental approaches, we conceptualize this process in this Review as a four-stage persistence continuum (Figure 1). The individual processes represented within this continuum are well established in the persistence literature; their organization into four conceptually distinguishable stages is introduced here as an analytical framework for examining which aspects of persistence are accessible to different experimental methods. The four stages comprise:

(i) Pre-antibiotic physiological state—the physiological characteristics of a cell before antibiotic exposure, including growth rate, metabolic activity, stress-response status, intracellular energy level, and other factors that may influence the probability of survival.
(ii) Survival during antibiotic exposure—the ability of a cell to withstand bactericidal antibiotic treatment without sustaining irreversible damage that prevents subsequent recovery.
(iii) Recovery after antibiotic removal—restoration of cellular functions required to resume growth, including repair of antibiotic-induced damage and reactivation of essential cellular processes.
(iv) Clonal expansion—the resumption of cell division resulting in population reconstitution, thereby providing experimental evidence that survival was reversible and productive.

These stages are separated for analytical purposes, although the underlying cellular processes may overlap in time. A cell may exhibit physiological features associated with persistence yet fail to survive antibiotic treatment. Conversely, another cell may survive treatment but fail to recover and proliferate. Within the framework proposed here, only cells that complete all four stages satisfy the established operational definition of a persister cell, which requires antibiotic survival followed by recovery and renewed proliferation without heritable resistance [3].

The key question is not simply whether a cell is a persister, but at which stage of the persistence continuum it is being observed. No single experimental approach captures the entire persistence continuum in its full biological complexity. Rather, each method provides access to particular stages or aspects of the process and therefore defines persister cells using assay-specific operational readouts. Consequently, different experimental approaches offer complementary rather than equivalent views of persistence. Recognizing which stages of the continuum and which cellular processes are accessible to a given method is therefore essential for correctly interpreting the biological conclusions drawn from persistence studies.

This conceptual framework provides the basis for the following sections, which examine how different experimental approaches interrogate distinct stages of the persistence continuum. As progressively stronger experimental evidence becomes available, the experimental designation assigned to bacterial cells also changes. Accordingly, the terminology used throughout this Review is summarized in Table 1.

In this Review, we do not use dormancy as a synonym for persistence. Dormancy refers to a physiological state that has historically been closely associated with the persister phenotype, particularly because early models frequently linked persistence to non-growing or dormant cells [14,15]. However, subsequent single-cell studies demonstrated that persisters can arise from both non-dividing and actively growing cells [4,5,16]. Accordingly, dormancy is considered here as a physiological state that may favor persistence under particular conditions, but not as a distinct cell designation within the persistence continuum or a defining feature of the persister phenotype.

## 3. Turning Persistence into a Measurable Phenomenon: The CFU Assay

The first experimental observations of persistence emerged from the colony-forming unit (CFU) assay, a well-established microbiological technique originally developed to quantify viable bacteria. CFU assays provided the experimental framework for Bigger’s pioneering observations (1944), which demonstrated that a small fraction of bacterial cells survived antibiotic treatment despite remaining susceptible upon regrowth [17]. These observations established the operational definition of persister cells that remains in use today [1].

**Figure 2:**
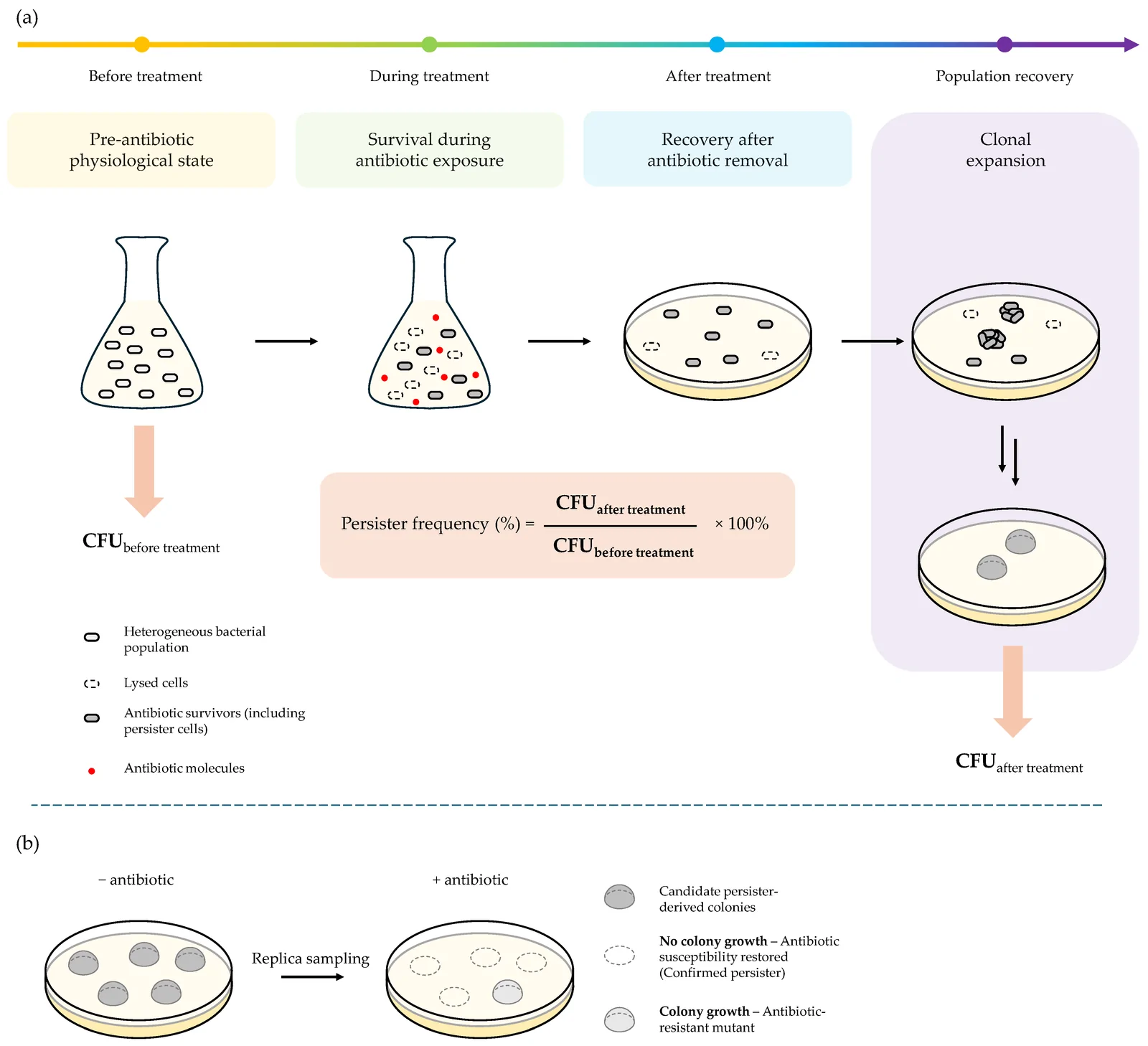
Colony-forming unit (CFU) assay used to quantify bacterial persistence. (**a**) Schematic workflow of the CFU assay, from antibiotic treatment to colony formation on solid medium. The persistence continuum is shown above the experimental workflow; only the clonal expansion stage is highlighted because colony formation is the sole direct readout of the CFU assay, whereas all preceding stages remain experimentally inaccessible. (**b**) Verification that persister-derived colonies are not antibiotic-resistant mutants by replica plating on antibiotic-containing medium. Colony growth identifies resistant mutants, whereas the absence of growth confirms restoration of antibiotic susceptibility (persister phenotype).

In a typical experiment, a bacterial culture is exposed to a bactericidal antibiotic under defined conditions (Figure 2a). Following treatment, the antibiotic is removed, and the culture is plated onto antibiotic-free agar after appropriate dilution. After incubation, visible colonies are counted, and each colony is assumed to originate from a single cell that survived treatment, recovered, and resumed proliferation. Persister frequency is therefore typically expressed as the ratio of CFU after antibiotic treatment to CFU before exposure (Figure 2a) [18,19]. To confirm that the resulting colonies represent persisters rather than resistant or heteroresistant mutants, recovered isolates must be retested for antibiotic susceptibility. Cells that retain the original minimum inhibitory concentration (MIC) and fail to grow in the presence of the selecting antibiotic are classified as persisters (Figure 2b) [1,2].

Within the persistence continuum, CFU assays provide access only to the final stage of the process—clonal expansion. They do not directly monitor the preceding stages of physiological state before antibiotic exposure, survival during treatment, or recovery after antibiotic removal. Instead, successful colony formation indicates retrospectively that all of these earlier stages have been completed. Consequently, CFU provides an operational measure of persistence rather than survival alone, making it the reference method for defining persister cells [1,2].

**Figure 3:**
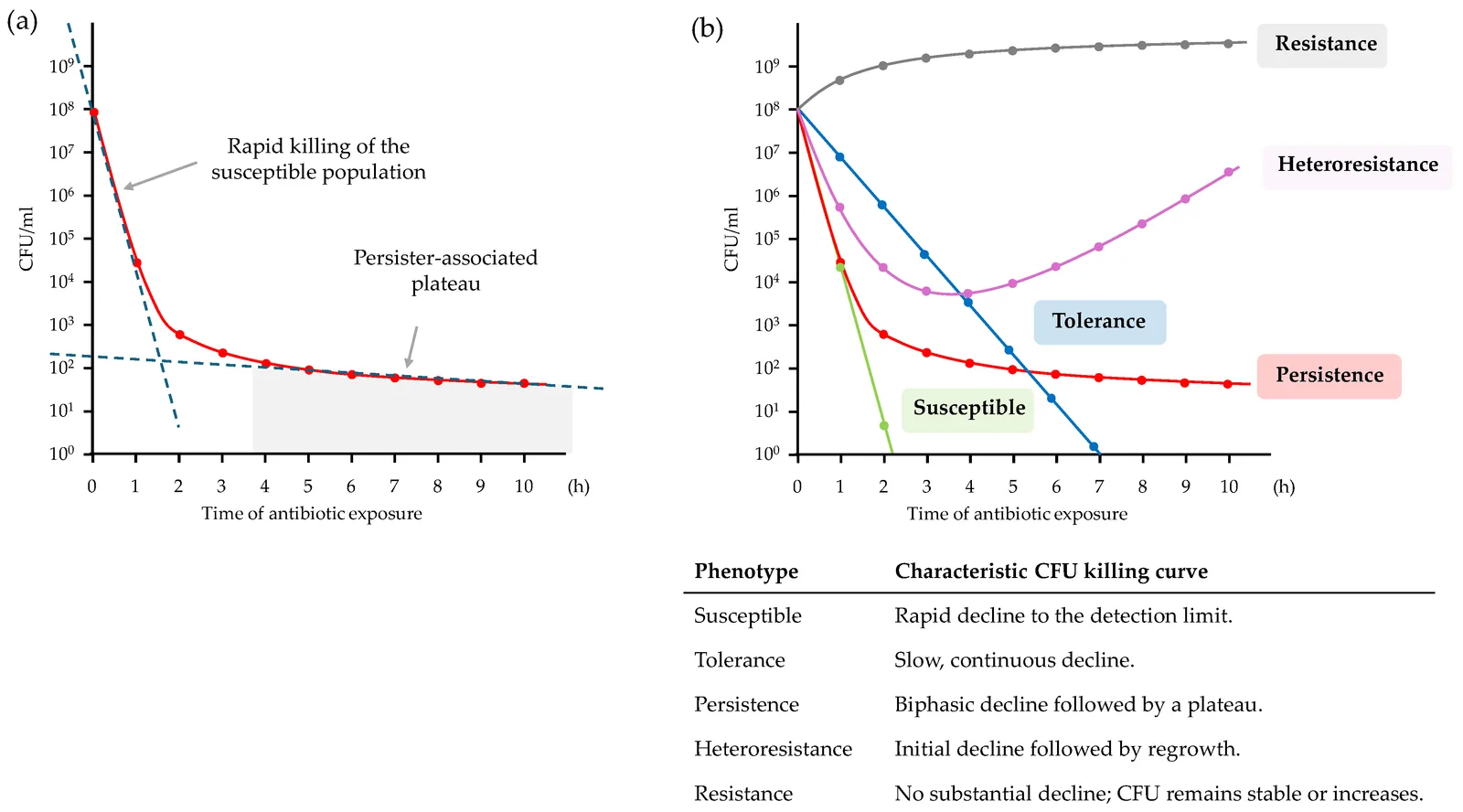
Antibiotic killing kinetics measured by CFU. (**a**) Killing curve of a susceptible bacterial population containing persister cells. The biphasic shape of the curve reflects two consecutive phases: an initial rapid decline in CFU caused by killing of the susceptible majority, followed by a much slower decline or plateau corresponding to the surviving persister subpopulation. The two phases are indicated by dashed trend lines. The shaded area indicates the later phase of antibiotic exposure, during which the surviving population is enriched in persister cells [1,14,20]. (**b**) Representative CFU killing curves for different bacterial responses to antibiotic exposure [21,22,23,24,25].

When CFU measurements are taken at multiple time points during antibiotic treatment, the resulting killing curves reveal the kinetics of bacterial killing. Depending on the antibiotic mechanism of action and experimental conditions, bacterial killing curves frequently exhibit biphasic kinetics, with an initial rapid killing phase followed by a second phase of markedly slower decline or a plateau (Figure 3a). The reproducible appearance of biphasic killing curves soon became one of the defining experimental signatures of persistence [1]. Moreover, their characteristic shape provided a simple experimental framework for distinguishing persistence from antibiotic tolerance, heteroresistance, and genetic resistance (Figure 3b) [1,14,20,21,22,23,24,25]. Among these phenotypes, tolerance is particularly important to distinguish from persistence because both enhance survival during antibiotic exposure without increasing the MIC. The difference lies in how this survival is distributed across the population: in persistence, only a subpopulation survives prolonged antibiotic exposure, whereas in tolerance, the population as a whole is killed more slowly, with viable cells eventually declining over time [1].

A similar biphasic pattern can also be observed when bacterial survival is measured as a function of antibiotic concentration at a fixed treatment time [19,26]. In both time- and concentration-dependent killing assays, susceptible cells are eliminated rapidly before the killing curve reaches a plateau, where the remaining survivors predominantly represent the persister fraction. Importantly, accurate quantification of persisters requires measurements to be performed only after this plateau has been reached. Sampling too early may overestimate persister frequency because it includes not only persister cells but also susceptible bacteria that have survived transiently and would be eliminated by longer exposure or higher antibiotic concentrations. Thus, defining appropriate antibiotic concentrations and treatment durations is essential for reliable CFU-based quantification of persistence.

CFU established persistence as a measurable biological phenomenon rather than an anecdotal observation, laying the groundwork for systematic mechanistic studies. Its simplicity, low cost, and high reproducibility also made CFU the primary screening tool for identifying genetic, physiological, and environmental factors that influence persister formation. In these experiments, persister frequency is measured using the CFU assay and compared between experimental and control conditions, such as wild-type and mutant strains or treated and untreated cultures. Using this experimental framework, researchers demonstrated that persister frequency depends strongly on the population’s physiological state, increasing from very low levels during early exponential growth to substantially higher levels in late exponential phase, stationary-phase cultures, and biofilms [19,26]. These observations established growth phase as one of the earliest and most robust correlates of persistence and provided the first evidence that persister formation is linked to bacterial physiology rather than occurring randomly.

Automated colony appearance assays further extended the information that could be obtained from CFU measurements by demonstrating that colonies originating from genetically identical cells frequently appeared at different times, revealing substantial heterogeneity in awakening dynamics despite identical survival outcomes [27,28].

CFU assays are quantitative but remain subject to important limitations. Because serial dilution is required before plating, only a small fraction of the treated culture is ultimately analyzed. When persister frequency is extremely low, rare survivors may be absent from the plated aliquot. Consequently, the probability of detecting persisters depends not only on their abundance but also on the volume of culture sampled. Moreover, CFU measurements inherently depend on the ability of surviving cells to form visible colonies under laboratory culture conditions. This limitation is particularly relevant for slow-growing or physiologically specialized organisms, including *Mycobacterium tuberculosis*, in which differentially culturable cells have been extensively described, and small-colony variants of *Staphylococcus aureus*, whose slow growth may delay or complicate colony detection under standard laboratory conditions, as well as certain intracellular pathogens [29,30,31,32,33,34,35].

CFU assays provide little insight into the biology of persistence. They identify which cells survived and resumed growth, but offer no information about their physiological state before antibiotic exposure, their behavior during treatment, or the mechanisms that enable recovery. Individual cells may differ profoundly in growth rate, metabolic activity, stress responses, chromosome replication status, or intracellular energy levels before antibiotic treatment, yet these differences remain invisible when persistence is assessed solely by colony formation. Addressing these questions required methods capable of measuring bacterial physiology rather than simply counting surviving cells.

## 4. Linking Physiology to Persistence: Population-Based Analyses

Population-based (“bulk”) analyses provided the first opportunity to investigate the physiology of persistence. Researchers compared bacterial populations that differed in persister abundance after genetic or environmental perturbations previously identified by CFU assays. The perturbed populations were analyzed alongside their untreated or wild-type controls using transcriptomic, proteomic, metabolomic, biochemical, and other population-based approaches to identify molecular characteristics associated with altered persistence.

**Figure 4:**
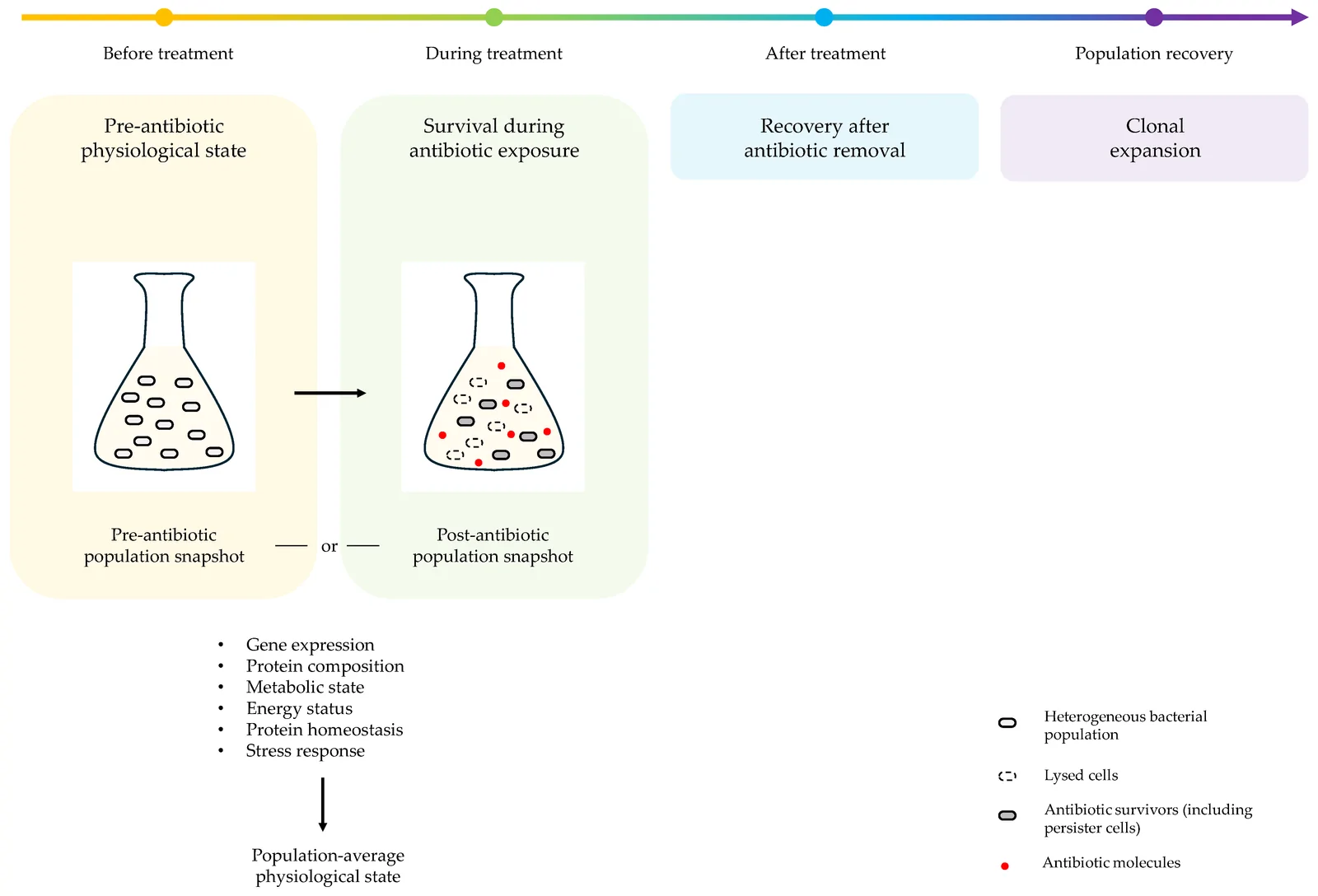
Population-based (bulk) analyses of bacterial persistence. Samples can be collected either before antibiotic exposure or after antibiotic treatment, and each analysis yields a single snapshot of the population. Multiple molecular and physiological approaches can then be used to characterize the sampled population. Bulk analyses measure the population’s average physiological state and do not provide information about individual cells or their histories.

Within the persistence continuum, bulk analyses primarily examine physiological states before and after antibiotic treatment (Figure 4) while relating these measurements to the final outcome determined by CFU. Rather than identifying persisters directly, they associate changes in population physiology with differences in persister abundance.

Bulk analyses fundamentally changed persistence research by generating the first mechanistic model explaining antibiotic survival. For the first time, persistence could be investigated as a physiological phenomenon rather than simply quantified using CFU assays. Population-based studies linked altered persister abundance to changes in global gene expression [19,36,37,38], toxin–antitoxin systems [39,40], cellular energetics and adenosine triphosphate (ATP) depletion [6,41], respiration and redox homeostasis [42,43], stringent response signaling [44,45,46], protein aggregation and dormancy depth [12,47,48,49,50], as well as global changes in the proteome [51,52,53,54]. These studies shifted persistence research from identifying antibiotic survivors to proposing physiological mechanisms that underlie persistence.

Population-based analyses also introduce an important conceptual limitation. They characterize heterogeneous bacterial populations rather than individual cells. Consequently, the measured transcriptome, proteome, metabolome, or ATP level represents the average physiological state of the entire population rather than the molecular state of any specific subpopulation. Bulk measurements therefore cannot determine whether an observed signal reflects the entire population or only one of several physiologically distinct subpopulations. Extreme phenotypes are averaged into a single value, revealing the overall trend while obscuring the underlying cellular heterogeneity. This limitation is particularly important in persistence research because bacterial populations analyzed by bulk methods are themselves physiologically heterogeneous. Depending on the experimental design, they may contain mixtures of actively growing cells, dormant cells, dead cells, viable but non-culturable (VBNC) cells, and, in antibiotic-based isolation protocols, cells whose physiology has already been altered by the treatment. Consequently, the measured physiological profile cannot be unambiguously assigned to naturally occurring persister cells within the heterogeneous population [55].

Despite these limitations, population-based analyses generated the first mechanistic hypotheses explaining antibiotic survival and transformed persistence from an observational phenomenon into a physiological problem. However, they could not identify which individual cells exhibited the measured characteristics, when these characteristics emerged, or whether they directly caused persistence. Testing these hypotheses therefore required methods capable of observing individual bacterial cells. This realization marked the transition from population-based analyses to single-cell approaches.

## 5. Capturing Persister Recovery at the Single-Cell Level: Time-Lapse Microscopy on Agarose Pads

The transition from population-based analyses to single-cell microscopy enabled the examination of individual bacterial cells rather than as part of a heterogeneous population. One of the simplest experimental approaches was time-lapse microscopy of bacteria immobilized on nutrient-containing agarose pads, allowing the same individual cells to be monitored continuously after antibiotic treatment. Because the same cells can be imaged continuously for many hours or even days, agarose pads became particularly useful for studying persisters, which are both rare and often resume growth only after prolonged lag periods [56]. Rather than recording persistence as a single experimental endpoint, this approach revealed that recovery itself is a dynamic cellular process.

**Figure 5:**
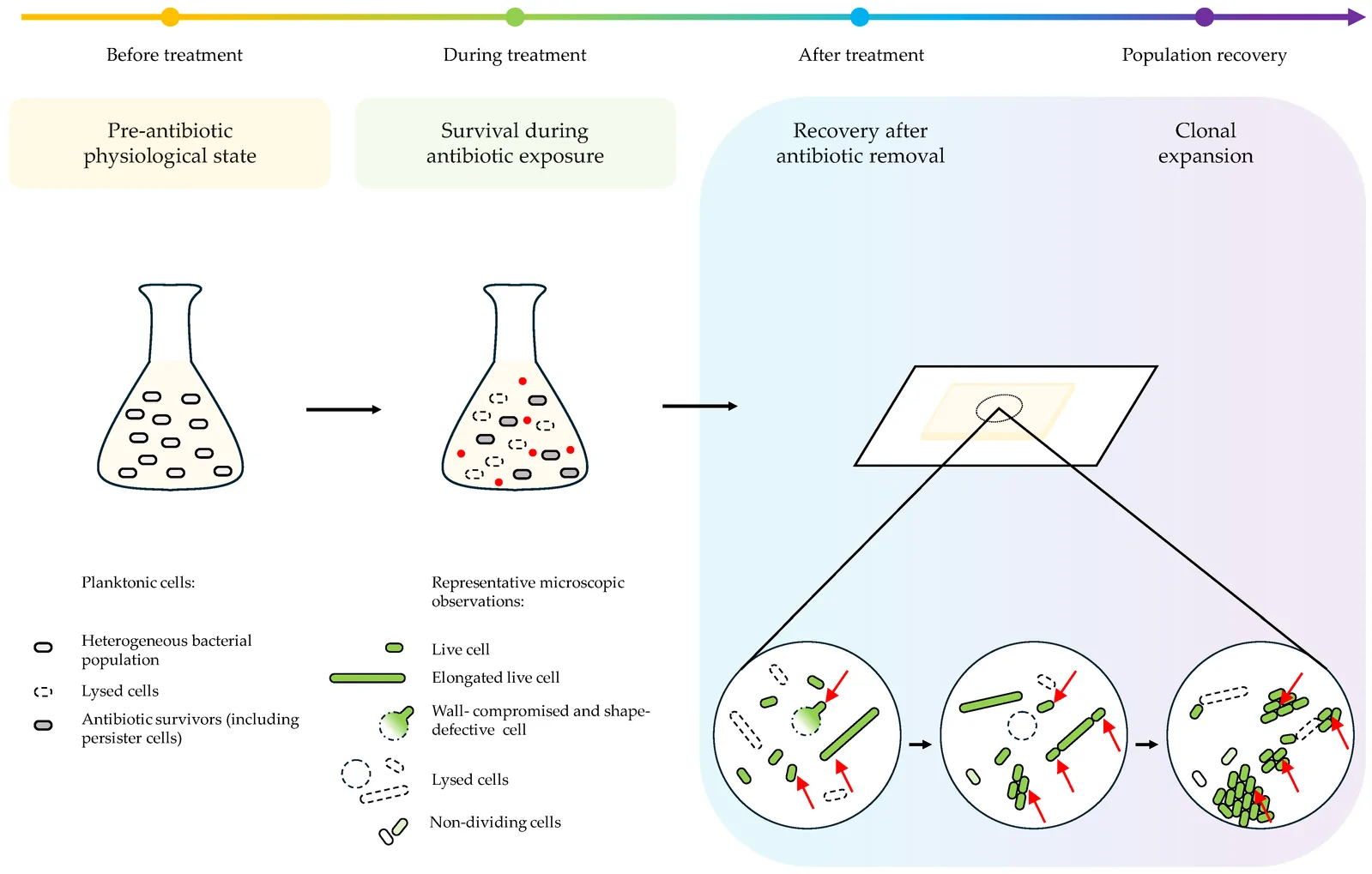
Time-lapse microscopy on agarose pads to observe post-antibiotic recovery at the single-cell level. Bacterial populations are transferred onto agarose pads after antibiotic treatment and monitored by time-lapse microscopy. This approach captures recovery and early clonal expansion, including cell morphology, awakening dynamics, first divisions, and heterogeneous recovery outcomes. Red arrows indicate cells identified retrospectively as persisters after successful growth resumption.

Time-lapse agarose-pad microscopy is conceptually straightforward (Figure 5). Following antibiotic treatment and drug removal, bacterial cells are transferred onto a thin, nutrient-containing agarose pad on a microscope slide or glass-bottom dish, then covered with a coverslip. Images are acquired repeatedly over time, allowing the same individual cells to be monitored as they remain non-dividing, resume growth, divide, or fail to recover [56,57]. Importantly, cells are not identified as persisters at the beginning of the experiment but only retrospectively, after prolonged observation reveals which surviving cells successfully resume proliferation. The recorded image sequence can then be reviewed retrospectively to reconstruct the recovery dynamics of cells identified as persisters. Depending on the experimental objective, recovery can be visualized using bright-field [11,58] or phase-contrast microscopy [16,43] to monitor cell morphology and division, or fluorescence microscopy to continuously track individual cells and their progeny [57].

Unlike CFU-based assays, which provide only the final outcome of survival, agarose-pad microscopy reconstructs the entire recovery process of individual surviving cells. Within the persistence continuum, this approach primarily captures the recovery and clonal expansion stages, allowing surviving cells to be followed from the moment antibiotic pressure is removed until they either resume growth or remain non-culturable (Figure 5).

Direct observation revealed that recovery is markedly heterogeneous, even among genetically identical bacteria under identical conditions. Time-lapse microscopy enabled quantitative measurements of lag time before the first division, cell elongation, filamentation, lysis, asymmetric partitioning of cellular contents, and subsequent microcolony formation [57]. Single-cell analyses further revealed multiple awakening phenotypes, ranging from immediate growth resumption to prolonged dormancy or complete failure to divide [6,11,57]. Importantly, cells with higher ribosome levels generally resumed growth more rapidly, providing one of the first direct links between the physiological state of cells later identified as persisters and their awakening kinetics [11]. Other studies proposed that recovery follows an exponential rather than purely stochastic pattern and also depends on antibiotic dose and intracellular efflux during resuscitation [57].

Because cells are observed on a static agarose surface, prolonged imaging is progressively complicated by colony overgrowth, cell clustering, and progeny accumulation, which can obscure cells that recover only after prolonged lag periods [7,56,59]. Patterned agarose pads improve cell separation and increase imaging throughput [59], but they do not overcome the approach’s principal methodological limitation. Environmental conditions cannot be dynamically controlled: nutrients and signaling molecules cannot be exchanged rapidly, antibiotics cannot be added or removed instantaneously, and cells cannot be continuously followed across changing environments. As a result, agarose-pad microscopy is used predominantly to investigate events occurring after antibiotic treatment and therefore captures only the recovery segment of the persistence continuum. The preceding stages—the cell’s physiological state before antibiotic exposure, its growth history, and the processes determining whether it would ultimately be identified as a persister—remain inaccessible. Consequently, agarose-pad microscopy revealed in unprecedented detail how cells identified retrospectively as persisters awaken, yet it could not explain why those particular cells, rather than their genetically identical neighboring cells, entered the persister state in the first place. Addressing this missing part of the persistence continuum required methods capable of continuously tracking the same individual bacterium before, during, and after antibiotic exposure under precisely controlled environmental conditions.

## 6. Reconstructing Cellular Histories: Microfluidics and Lineage Tracking

**Figure 6:**
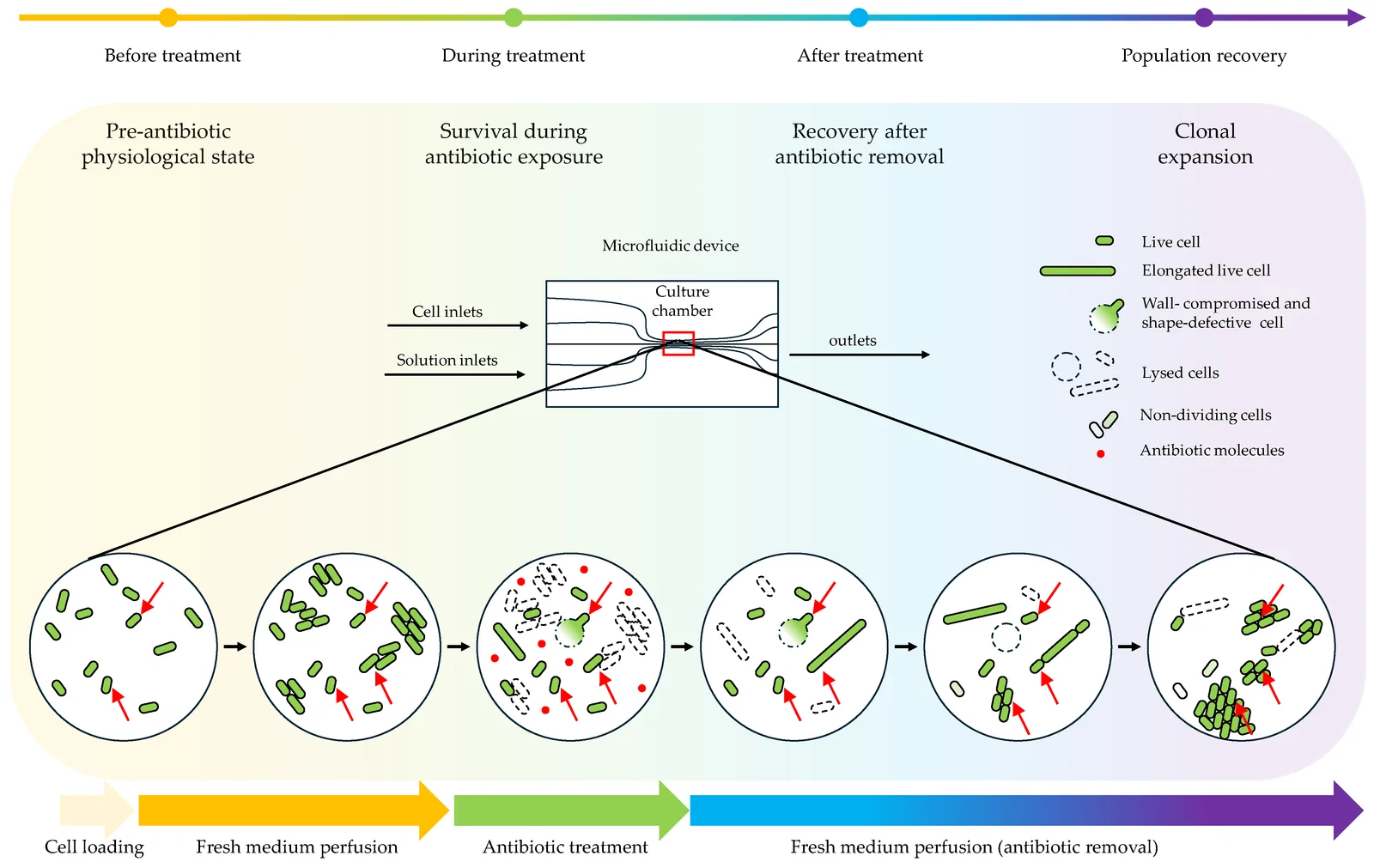
Microfluidic platforms enable continuous single-cell tracking throughout the persistence continuum. Bacterial cells are loaded into a microfluidic device, equilibrated under continuous perfusion with fresh medium, exposed to antibiotic-containing medium, and monitored after antibiotic removal. Continuous observation enables reconstruction of cellular histories, including pre-treatment physiology, survival, morphological changes, recovery dynamics, and early clonal expansion. Red arrows indicate cells identified retrospectively as persisters after successful growth resumption.

The transition from agarose-pad microscopy to microfluidic platforms represented an important methodological advance by extending observation across the entire persistence continuum (Figure 6). As in agarose-pad microscopy, cells are identified as persisters only retrospectively, after surviving antibiotic treatment and resuming growth. However, because the same individual cells are continuously monitored throughout the entire experiment, microfluidic platforms enable reconstruction of their complete cellular histories, including pre-treatment growth behavior, responses during antibiotic exposure, awakening dynamics following antibiotic removal, and subsequent clonal expansion.

Unlike agarose-pad microscopy, where environmental conditions remain essentially static, microfluidic systems enable rapid and precisely timed changes in the extracellular environment. Bacterial cells are confined within narrow channels or microchambers while fresh medium, antibiotics, or other environmental stimuli are continuously supplied, keeping cells in a fixed focal plane and enabling uninterrupted long-term imaging. Consequently, individual cells can be exposed sequentially to different experimental conditions while their growth, morphology, and recovery are monitored continuously throughout the experiment.

Different microfluidic architectures have been developed for this purpose, including groove-based devices [18,60], mother-machine platforms [6,7,61,62], multi-cell chambers [5,63,64] and single-cell microchambers [4]. These platforms differ not only in engineering design but also in the biological information they provide. Groove-based devices and mother-machine platforms are particularly well suited for reconstructing lineage relationships and monitoring cell growth and division because individual bacteria remain spatially separated throughout the experiment. In contrast, chamber-based platforms permit more comprehensive observation of cellular morphology, enabling visualization of events such as filamentation, asymmetric partitioning, and other morphological transitions. Although these platforms differ in engineering design, each provides a distinct window into persister biology, emphasizing different cellular behaviors and therefore different stages or aspects of the persistence continuum.

This approach enabled several long-standing hypotheses about persister formation to be tested directly. Early studies suggested that persisters predominantly arose from pre-existing slow-growing or dormant cells [14,15]. However, subsequent lineage-tracking experiments demonstrated that pre-treatment growth rate alone does not universally predict persistence. Depending on the bacterial species, growth conditions, and antibiotic used, persisters may arise from both slowly growing and actively proliferating cells [4,5]. Instead, continuous single-cell observations showed that multiple physiological trajectories can lead to the persister phenotype.

The consequences of these diverse trajectories were most apparent during the recovery phase. Microfluidic studies confirmed the heterogeneous recovery patterns previously observed with agarose-pad microscopy and placed these trajectories in the context of each cell’s earlier physiological history. Cells that ultimately survived antibiotic treatment exhibited markedly different lag times, recovery kinetics, and morphological transitions despite experiencing identical environmental conditions [4,6]. Importantly, the interpretation of these recovery trajectories depended strongly on the antibiotic’s mechanism of action [3]. For example, survival after fluoroquinolone treatment did not necessarily predict successful regrowth because cells first had to repair extensive antibiotic-induced damage before division resumed [5,64,65], whereas recovery after β-lactam treatment was largely determined by morphological events such as filamentation and asymmetric partitioning during subsequent divisions [4]. These observations demonstrated that survival and recovery represent distinct stages of the persistence continuum, and their relative importance depends on the antibiotic mechanism.

Integrating fluorescent biosensors into microfluidic imaging enabled the association of specific physiological processes with the subsequent fate of individual cells. Reporter-based studies showed that cells that were later identified as persisters often differed from susceptible cells before antibiotic exposure, though no universal physiological signature emerged. For example, cells with reduced intracellular ATP were more likely to survive ampicillin treatment in one study, consistent with a low-energy model of persistence [6], whereas another live-cell imaging study found no correlation between ATP levels and persistence in a stringent response alarmone (p)ppGpp-dependent system [44]. Similarly, cells later identified as persisters exhibited lower intracellular pH before antibiotic exposure, a phenotype dependent on tryptophanase activity [66]. Fluorescent reporters also enabled direct testing of candidate molecular mechanisms of persistence. For example, reporter-based single-cell measurements failed to confirm the proposed relationship between activation of type II toxin–antitoxin systems and persistence, as reporter fluorescence did not predict which cells subsequently resumed growth after antibiotic treatment [44]. Together, these studies indicate that no single physiological parameter explains persistence across all experimental conditions. Consequently, no individual reporter provides a comprehensive picture of persister physiology. Instead, each biosensor illuminates only one physiological dimension of the persistence continuum, and biological conclusions remain constrained by the physiological process being monitored.

A practical limitation of all single-cell persistence studies is the extreme rarity of naturally occurring persisters, which typically constitute only 10^−4^–10^−6^ of exponentially growing bacterial populations [19]. Consequently, even microfluidic experiments that monitor tens or hundreds of thousands of cells often identify only a handful of true persisters. For example, Goormaghtigh and Van Melderen (2019) detected only six persisters among approximately 47,000 *Escherichia coli* cells exposed to ofloxacin [5], Manuse et al. (2021) identified sixteen survivors following ampicillin treatment [6], and Umetani et al. (2025) recovered only six persisters from approximately 3.8 × 10^5^ exponentially growing cells [4].

The main consequence of these small sample sizes is limited confidence in how representative the observed physiological trajectories are of the broader persister population. Physiological characteristics observed in only a few captured persisters may represent common features of persistence, but they may also reflect rare individual trajectories or stochastic variation. Increasing the number of retrospectively identified persisters is therefore essential for distinguishing reproducible physiological associations from features observed only in individual cells.

The small number of persisters captured in individual experiments also poses a significant reproducibility challenge. No universally accepted minimum number of persister cells has been established for single-cell persistence studies, and an arbitrary numerical threshold would be difficult to justify because the required sample size depends on the biological question, effect size, and variability of the measured phenotype. Reproducibility should therefore rely on independent biological experiments and transparent reporting of the total number of cells analyzed, the number of retrospectively confirmed persisters, their distribution across independent experiments, and the criteria used for persister identification. Reporting the variability of the measured phenotype among individual persister cells is also important for assessing the consistency of observed associations across experiments.

## 7. Increasing the Probability of Studying Persisters: Strategies for Population Enrichment

An alternative strategy for overcoming the rarity of naturally occurring persisters was to increase their relative abundance within the bacterial population before downstream analyses. Several experimental approaches have been used for this purpose, including naturally occurring physiological states associated with elevated persister frequencies, genetic models, and transient chemical perturbations that promote entry into persister-enriched states (Table 2). In this context, the term enrichment refers to increasing the experimental probability of detecting persisters rather than implying equivalent biological mechanisms across these diverse approaches.

The earliest enrichment approaches took advantage of physiological states associated with higher persister frequency, particularly stationary phase and biofilm growth. Keren et al. (2004) demonstrated that persister frequency increases markedly upon entry into stationary phase [19], making stationary-phase cultures an attractive source of persister-enriched populations for downstream physiological and molecular studies, including investigations of metabolic regulation, protein aggregation, respiratory activity, and stress responses associated with persistence (Table 2). Similarly, biofilm growth has been associated with substantially higher persister frequencies than planktonic cultures, providing persister-enriched populations that have been used to investigate persistence in structured bacterial communities (Table 2). Using this approach, Miyaue et al. (2017) further showed that biofilm-derived persisters retained elevated antibiotic tolerance for prolonged periods after transfer to fresh nutrient-rich medium, suggesting the existence of a physiological “memory effect” established during biofilm growth [67]. More recently, prolonged starvation protocols have also been used to increase the proportion of persister cells, and Vedelaar et al. introduced a nutrient-shift protocol in which an abrupt change in carbon source transiently induces bacterial dormancy, thereby generating persister-enriched populations [68]. However, none of these physiological approaches selectively enrich for persisters. Instead, they are heterogeneous mixtures of actively growing cells, slow-growing cells, damaged cells, viable but non-culturable (VBNC) bacteria, and persisters. Consequently, the increased abundance of persisters is accompanied by greater physiological heterogeneity, complicating the interpretation of downstream analyses.

A fundamentally different strategy increases persister abundance genetically. High-persistence (*hip*) mutants, particularly the *hipA7* allele, increase the probability that bacterial cells enter persistence [40,69]. Before naturally occurring persisters could be routinely investigated at the single-cell level, *hipA7* strains became one of the principal experimental models for persistence research, enabling pioneering studies of single-cell awakening as well as later investigations of ribosome organization, transcriptional programs, and metabolic quiescence (Table 2). Nevertheless, genetic enrichment does not increase naturally occurring persisters. Instead, it enriches a genetically altered physiological state whose properties may not fully represent the diversity of persistence mechanisms operating in wild-type populations.

A third strategy relies on transient pharmacological perturbation immediately before bactericidal antibiotic treatment. Several compounds that transiently inhibit bacterial growth or essential cellular processes, including rifampicin, tetracycline, and carbonyl cyanide *m*-chlorophenyl hydrazone (CCCP), have been shown to markedly increase survival following subsequent bactericidal antibiotic treatment [70]. Together, these compounds established the principle that transient pharmacological perturbation can be exploited as an enrichment strategy. Chemical enrichment strategies have subsequently been extended to *S. aureus* and *Pseudomonas aeruginosa* [71,72,73], demonstrating that chemically induced enrichment is applicable across multiple bacterial species (Table 2).

Although several different compounds have been explored experimentally, rifampicin pretreatment has become the most widely adopted chemical enrichment strategy in subsequent persistence studies. By briefly inhibiting transcription, rifampicin markedly increases the proportion of cells surviving subsequent bactericidal antibiotic treatment. Antibiotic susceptibility is restored after regrowth, consistent with the non-heritable nature of the induced phenotype. In the original protocol developed by Kwan et al., exponentially growing cells were pretreated with rifampicin (100 µg/mL) for 30 min followed by rifampicin removal and subsequent bactericidal antibiotic challenge [70]. Rifampicin pretreatment has subsequently been applied as an enrichment strategy for proteomic analyses, single-cell microscopy, genetic screens, flow cytometry, and high-throughput anti-persister drug discovery (Table 2). Consequently, many of the current insights into persister physiology have been obtained using rifampicin-enriched populations. Because rifampicin deliberately alters bacterial physiology immediately before bactericidal antibiotic exposure, the physiological state subsequently analyzed is, at least in part, shaped by the enrichment procedure itself.

Importantly, these pharmacological pretreatments are not biologically neutral. Under representative enrichment conditions, rifampicin, tetracycline, and CCCP themselves reduce population viability to varying extents [70] (Table 2), such that only a fraction of the initial population proceeds to the subsequent bactericidal antibiotic challenge. Consequently, chemical enrichment involves not only transient physiological reprogramming but also an initial selection step that further shapes the composition of the population entering the subsequent antibiotic challenge.

Pretreatment concentration and duration are important experimental variables that influence the efficiency of chemical enrichment. In the original study by Kwan et al., these parameters were optimized separately for rifampicin, tetracycline, and CCCP, and different pretreatment conditions resulted in different levels of subsequent antibiotic survival [70]. The available evidence, however, does not establish whether differences in pretreatment conditions generate qualitatively distinct persister populations. Therefore, the specific conditions used for chemical enrichment should be considered when comparing enriched populations and interpreting their downstream physiological or molecular characteristics.

**Table 2:** Experimental strategies for persister enrichment and representative applications in persistence research.

Enrichment Strategy	Representative Approach	Biological Basis	Typical Post-Treatment Survival *		Representative Applications	Main Limitation
Physiological	Stationary-phase cultures	Nutrient limitation and growth arrest	~1% [19]	[36,43,45,47,74]	Metabolic slowdown; protein aggregation; stringent response; respiration; environmental adaptation	Persisters remain embedded within highly heterogeneous populations containing VBNC, damaged, dormant and actively growing cells.
	Biofilm cultures	Nutrient and oxygen gradients	Up to ~10% [67]	[67,75,76]	Spatial heterogeneity; metabolic gradients; biofilm-associated persistence; host adaptation	
	Nutrient shift	Carbon source transition	~50–100% [68,77]	[76,77,78]	Metabolic reprogramming; σS-mediated stress response; metabolic flux limitation	
Genetic	*hipA7* mutant	Genetically induced growth arrest	Up to ~10% [40,69]	[18,79,80,81,82]	Growth arrest; toxin–antitoxin regulation; awakening dynamics	Artificial genetic background may not reflect wild-type physiology.
Chemical	Rifampicin	Transient transcription inhibition	~60–70% [70]	[11,52,58,73,80,83,84,85,86,87,88,89,90,91]	Transcriptional reprogramming; ATP homeostasis; ribosome hibernation	The enrichment treatment itself reshapes bacterial physiology; different compounds enrich distinct physiological states.
	Tetracycline	Transient translation inhibition	~20–40% [70]	[84,85]	Translation regulation; ribosome hibernation; persister resuscitation	
	CCCP	Collapse of proton motive force	~60–75% [70]	[71,72,84]	Energy depletion; membrane energetics; ATP homeostasis; ribosome hibernation	

* Approximate percentages of the initial bacterial population surviving subsequent antibiotic exposure. These values represent survivor fractions rather than direct measurements of spontaneous persister frequency. Values depend strongly on bacterial strain, enrichment conditions (including compound concentration and pretreatment duration), and the type, concentration, and duration of the subsequent bactericidal antibiotic treatment. CCCP—carbonyl cyanide *m*-chlorophenyl hydrazone; VBNC—viable but non-culturable.

Although these strategies differ in implementation, they share an important conceptual limitation. None enriches persisters without simultaneously selecting or modifying bacterial physiology. Stationary phase favors cells adapted to nutrient limitation, genetic models increase persistence through defined molecular pathways, and chemical pretreatments transiently reshape cellular physiology before antibiotic exposure. Enrichment strategies therefore produce distinct persister-enriched populations whose physiological characteristics depend on the underlying enrichment method. Thus, enrichment acts not only as a technical tool for increasing persister abundance, but also as a biological filter determining which persister subpopulation becomes available for subsequent analysis. As a result, conclusions drawn from transcriptomic, proteomic, metabolomic, or single-cell studies reflect not only the analytical method employed, but also the enrichment strategy used beforehand. The physiological properties identified in these populations should therefore be interpreted as characteristics of experimentally generated persister-enriched populations rather than as universal properties of naturally occurring persisters. Moreover, enrichment increases the frequency of persisters without eliminating the overwhelming majority of non-persister cells. Consequently, downstream analyses remain influenced by population heterogeneity, leaving unresolved the challenge of studying naturally occurring persister cells in isolation.

## 8. Isolating Candidate Persister Cells: Sorting and Selection Strategies

Enrichment strategies generate persister-enriched populations but do not physically separate candidate persister cells from the surrounding bacterial population. Consequently, downstream physiological, biochemical, or molecular analyses are still performed on heterogeneous mixtures containing actively growing, dormant, dead, and damaged bacteria. This limitation stimulated the development of sorting and selection approaches designed to enrich candidate persister cells for downstream analyses (Table 3).

Because persistence is operationally defined by survival after antibiotic treatment, the earliest isolation strategies exploited this defining characteristic directly. Persisters can be identified only retrospectively by demonstrating survival, recovery, and regrowth after antibiotic exposure, making antibiotic treatment itself the first selection step. Cells that remain intact after treatment are then separated from lysed bacteria and cellular debris for further analysis. One of the earliest isolation strategies employed ampicillin, which selectively lyses actively growing cells while leaving intact survivors that can be separated from lysed cells and debris by centrifugation or filtration [19]. A conceptually related strategy exploited the differential morphological response to β-lactam antibiotics. During cephalexin treatment, susceptible bacteria undergo extensive filamentation, whereas a small fraction of non-filamenting cells remains short and can be isolated by filtration [7]. Both approaches isolate cells that have already demonstrated antibiotic tolerance and therefore provide valuable cell populations for post-treatment physiological characterization. However, subsequent studies demonstrated that these isolated fractions are not composed exclusively of persisters but also contain substantial numbers of viable but non-culturable (VBNC) cells, which may even outnumber persisters depending on treatment conditions [93,94]. Moreover, because these strategies isolate cells only after antibiotic exposure, they provide little information about the physiological state preceding persistence and therefore cannot explain why only a subset of genetically identical cells ultimately survives treatment and resumes proliferation.

Addressing this question requires prospective sorting of candidate persister cells before antibiotic exposure. The major challenge, however, is that cells that later prove to be persisters cannot be recognized prospectively. Unlike many specialized cell types, persisters lack a universal molecular or genetic marker that enables their prospective identification. Consequently, prospective isolation strategies rely on surrogate physiological characteristics that are assumed to increase the probability that a given cell will subsequently be identified as a persister. For this reason, all prospective sorting strategies ultimately require post hoc confirmation that the isolated cells fulfilled the operational definition of persistence.

One of the earliest biological assumptions was that persisters predominantly arise from non-growing or slowly growing bacteria [14,19]. This hypothesis led to the development of fluorescence-dilution systems, in which a stable fluorescent protein is first accumulated during an induction period and subsequently diluted among daughter cells after cell division. After removal of the inducer, dividing cells progressively lose fluorescence, whereas non-dividing cells retain a high fluorescent signal and can be isolated by fluorescence-activated cell sorting (FACS) [92]. Although non-dividing populations were consistently enriched for candidate persister cells, subsequent studies demonstrated that persisters may also originate from actively growing bacteria [16], indicating that growth arrest is a probabilistic rather than universal characteristic of persistence.

Subsequent studies proposed that persistence might be more closely linked to metabolic activity than to growth itself. Reporter systems using unstable fluorescent proteins placed under the control of the ribosomal promoter *rrnB* P1 enabled the isolation of bacterial cells with reduced ribosomal promoter activity, a proxy for low metabolic activity [36]. Later work demonstrated that reduced reporter activity closely mirrored intracellular ATP depletion, supporting the concept that cellular energy state contributes to antibiotic tolerance [41]. Nevertheless, as with growth arrest, reduced metabolic activity represents a physiological state associated with an increased probability of persistence rather than a reliable predictor or universal feature of persister cells.

Initial prospective isolation strategies therefore focused on global physiological characteristics presumed to predispose cells to persistence before antibiotic exposure. More recently, however, attention has shifted towards monitoring physiological pathways associated with the response to specific classes of antibiotics and the subsequent recovery process. One example is the use of fluorescent SOS reporters to investigate heterogeneity in the DNA damage response after fluoroquinolone treatment [95]. Unlike growth arrest or metabolic activity, which are constitutive physiological states, SOS reporters monitor a repair pathway activated in response to antibiotic-induced DNA damage. These reporters distinguish bacterial subpopulations according to their capacity to repair DNA and resume growth after fluoroquinolone removal. Such approaches therefore provide insight primarily into mechanisms governing post-antibiotic recovery and population re-establishment. Regardless of the physiological marker employed, FACS-based isolation introduces additional methodological constraints. Reporter performance, sorting conditions, and post-sort viability may influence the composition of the recovered population, requiring careful validation to ensure that the analyzed cells faithfully represent the selected subpopulation before biological conclusions are drawn.

Although these approaches have substantially expanded our ability to investigate rare bacterial subpopulations, their interpretation requires particular caution. None of these strategies specifically isolates persister cells. Each method selects bacteria based on a predefined physiological characteristic or operational criterion that increases the probability of persistence but does not uniquely identify the persister phenotype. Sorted populations should therefore be interpreted as populations enriched for candidate persister cells; individual cells can be confirmed as persisters only after demonstrating antibiotic survival followed by recovery and renewed proliferation without heritable resistance. This distinction is particularly important for physiological characteristics such as growth arrest or low metabolic activity, which are neither unique to nor universally present in persister cells. As a result, downstream physiological, biochemical, transcriptomic, or proteomic analyses describe the isolated subpopulation rather than a universally defined persister state.

## 9. Interpreting Persistence Measurements: The Importance of Experimental Context

Throughout this Review, we have shown that different experimental approaches interrogate distinct aspects of bacterial persistence. However, methodological differences alone cannot account for the remarkable variability often observed across studies. Even when identical techniques are used, measured persister frequencies may differ by several orders of magnitude because experiments are performed under different biological conditions. Persistence therefore depends not only on how it is measured, but also on the biological context in which it is measured. The physiological state of the bacterial population is shaped long before antibiotic exposure and continues to change throughout the experiment, influencing both survival and subsequent recovery.

One important determinant of this physiological state is the inoculum. Cultures initiated from old stationary-phase inocula generally show substantially higher frequencies of antibiotic survivors than those derived from freshly growing cells [96]. This difference reflects both the greater abundance of dormant and slow-growing cells and the population’s physiological history. Dormant cells present in the inoculum may survive repeated dilution and remain detectable during subsequent persistence assays. Consequently, some survivors recovered after antibiotic treatment may not represent persisters formed de novo during exponential growth, but rather dormant cells carried over from the inoculum itself [4,96,97].

The antibiotic challenge further shapes which bacterial cells survive and which physiological processes become relevant for persistence. β-lactams and fluoroquinolones provide two contrasting examples of how antibiotic mechanism determines which stages of the persistence continuum become most informative experimentally. β-lactam antibiotics such as ampicillin inhibit cell-wall synthesis, leading to extensive lysis of susceptible cells and making the distinction between lysed and intact cells directly observable during antibiotic exposure [79]. In contrast, fluoroquinolones primarily induce lethal DNA damage while largely preserving cell integrity. In this case, post-treatment survival cannot be interpreted independently of the subsequent recovery phase, during which DNA repair and restoration of proliferative capacity determine whether a surviving cell ultimately fulfills the operational definition of a persister [3,4,5]. Consequently, the timing of downstream analyses becomes antibiotic-dependent. For example, molecular profiling performed immediately after fluoroquinolone exposure may primarily capture antibiotic-induced damage responses, whereas analyses performed during recovery can reveal repair processes associated with successful resumption of growth.

The outcome of persistence assays is also influenced by the conditions under which antibiotic treatment is performed. Antibiotic concentration and exposure time affect killing efficiency [97]. Likewise, high-density cultures often show reduced killing and increased survival because of altered metabolism, nutrient depletion, accumulation of extracellular metabolites, and reduced effective antibiotic exposure at the level of individual cells [98]. These effects are particularly relevant in stationary-phase cultures, which combine the highest reported persister frequencies with the highest cell densities. Under such conditions, not all bacterial cells may experience the same effective antibiotic concentration or duration of exposure. As a result, increased survival may partly reflect heterogeneous antibiotic exposure across the population rather than differences in persister formation alone.

Much of the foundational work on bacterial persistence has relied on laboratory-adapted strains. Although such strains have been instrumental in identifying mechanisms associated with persister formation and survival, clinical isolates may display physiological properties, including a propensity for persistence, that differ substantially from those of laboratory strains [34,99,100,101,102,103]. During infection, bacterial populations face host-associated pressures and repeated antibiotic treatments that can progressively shape their persistence phenotype.

Longitudinal studies of *P. aeruginosa* isolated from patients with cystic fibrosis provide a particularly striking example. Mulcahy et al. showed that high-persister variants can emerge during chronic infection, with a late isolate exhibiting an approximately 100-fold higher persister frequency than a clonally related early isolate [104]. High-persister phenotypes were also common among late isolates obtained from additional patients and could not be explained solely by increased antibiotic resistance.

A complementary example comes from *S. aureus*, where patient-derived isolates exposed to host-associated conditions displayed greater antibiotic persistence than laboratory strains [105]. Moreover, genetically indistinguishable isolates recovered from the same patient exhibited pronounced physiological heterogeneity when examined directly after isolation, which was substantially reduced following cultivation in nutrient-rich laboratory medium. Considerable variation can also occur among independent clinical isolates of the same species. Among 37 clinical *Acinetobacter baumannii* isolates, for example, persister levels following polymyxin B treatment varied markedly between isolates despite their susceptibility to the antibiotic, with two isolates showing no detectable persister population under the conditions tested [103].

The importance of biological context becomes even more apparent when standardized in vitro conditions are compared with the complex environments encountered during infection. In the host, bacteria face nutrient limitation, variable oxygen availability, immune pressure, polymicrobial interactions, and other local conditions that differ across tissues and infection sites. This spatial and physiological heterogeneity influences bacterial growth, metabolism, stress responses, and antibiotic exposure, generating bacterial states that may be absent or poorly represented in conventional laboratory cultures.

Biofilms offer a way to introduce some of this environmental complexity while retaining the experimental control of an in vitro system. Their spatial organization generates local gradients in nutrient and oxygen availability, metabolic activity, growth rate, and antibiotic penetration, creating physiologically distinct bacterial subpopulations within the same culture [106,107]. Even within an in vitro system, introducing such environmental heterogeneity can substantially change the persistence phenotype observed experimentally. In a classical comparison of *P. aeruginosa* populations, Spoering and Lewis observed approximately 0.001% persisters following ofloxacin treatment in exponentially growing planktonic cultures compared with approximately 0.1% in biofilms [108]. Importantly, stationary-phase planktonic cultures contained an even larger persister fraction, reaching approximately 2.5%, indicating that the persistence phenotype associated with biofilms cannot be attributed to spatial organization alone but also reflects the physiological states generated by the local environment.

The influence of the local environment is also evident when bacteria are examined within host-associated systems. During macrophage infection, Helaine et al. identified a nonreplicating *Salmonella* population containing persisters whose formation was promoted by vacuolar acidification and nutritional deprivation [109]. Subsequent work further showed that persisters maintained in this intracellular environment remained transcriptionally, translationally, and metabolically active [110], illustrating that host-associated conditions can influence not only persister formation but also the physiological properties observed in persister cells.

The contribution of individual environmental factors can also differ when persistence is examined under conditions that more closely mimic those encountered during infection. Using infected mice together with tissue-mimicking chemostats, Fanous et al. systematically compared several conditions experienced by *Salmonella* during infection and found that nutrient starvation, through its restriction of bacterial replication, had a substantially greater impact on antibiotic survival than several other infection-associated stresses [111]. The relative contribution of individual factors to antibiotic survival therefore depended on the environmental conditions under which they were examined.

Because strain background, population history, and local environmental conditions can alter both the abundance and the physiological properties of persisters, these variables should be treated as integral components of persistence experiments and reported in sufficient detail to allow reproducibility and meaningful comparison across studies. Biological context and experimental methodology are therefore inseparable. Together, they determine which bacterial cells become available for analysis, which stages of the persistence continuum can be observed, and ultimately how persistence is interpreted. The major experimental filters that shape persister identification and biological interpretation are summarized in Table 4.

## 10. Extending Persistence Research Beyond Controlled In Vitro Systems

Controlled in vitro systems remain essential for dissecting the mechanisms underlying bacterial persistence. Their experimental simplicity allows independent manipulation of variables, precise control of antibiotic exposure, and monitoring of bacterial responses with temporal and, increasingly, single-cell resolution. In particular, time-lapse microscopy and microfluidic approaches can retrospectively link the physiological state of an individual cell before antibiotic exposure to its survival, recovery, and subsequent clonal expansion. Such experimental control is difficult to achieve in more complex biological systems. However, as discussed in Section 9, strain background and environmental conditions can substantially alter both persister frequency and physiology. Mechanisms identified under highly controlled conditions therefore cannot be assumed to have the same relative importance during infection [111,112].

Several methods used to study persistence in vitro can nevertheless be extended to host-associated systems, although the information they provide changes with increasing biological complexity. Culture-based approaches can quantify viable bacteria recovered from infected cells or tissues following antibiotic treatment, but they retain the fundamental limitation of conventional CFU assays: colony formation provides an endpoint measure of successful regrowth rather than a direct record of the physiological history of surviving cells. Fluorescence-based approaches can preserve substantially more information at the single-cell level. Helaine et al. adapted fluorescence dilution and single-cell analysis to macrophage infection to distinguish replicating from nonreplicating *Salmonella* and identify intracellular persisters [109]. Subsequent methodological developments have combined antibiotic survival assays, fluorescence dilution, flow cytometry, and host-cell infection to quantify and characterize persisters during infection [113,114].

Increasing biological complexity, however, progressively restricts some experimental capabilities that are particularly valuable for studying persistence. Continuous observation of the same bacterium before, during, and after antibiotic treatment is feasible in microscopy and microfluidic systems but becomes considerably more difficult within infected tissues or whole animals. Spatial localization, host–cell interactions, variable antibiotic exposure, and difficulties in recovering and re-identifying individual bacteria limit reconstruction of the complete cellular history required for unequivocal retrospective identification of a persister. Host-associated and in vivo models therefore provide greater physiological relevance but do not necessarily provide greater resolution of the persistence process. Rather than replacing controlled in vitro experiments, they provide a complementary level of evidence for determining whether mechanisms identified under simplified conditions remain relevant during infection [112].

The importance of integrating these levels of investigation is illustrated by recent work on *Salmonella* antibiotic survival under physiologically relevant conditions. Fanous et al. combined conventional CFU measurements with real-time single-cell analysis and found that the two approaches produced different views of bacterial killing [111]. Biphasic CFU kinetics were consistent with the presence of a highly resilient subpopulation, whereas real-time measurements indicated relatively uniform and slow antibiotic-induced damage across the population. Much of the apparent biphasic response arose from continued bacterial death during post-treatment recovery rather than from pronounced differences in damage during antibiotic exposure. This example illustrates that extending persistence research to physiologically relevant systems is not simply a means of validating an in vitro phenotype; combining different readouts can also change the biological interpretation of antibiotic survival.

Despite substantial progress in the experimental characterization of persistence, its direct impact on routine clinical decision-making remains limited. Unlike antibiotic resistance, persistence is not currently assessed with standardized diagnostic assays or clinically established breakpoints, and measurements remain highly sensitive to inoculum preparation, growth conditions, antibiotic exposure, sampling strategy, and recovery conditions [1,115,116]. Consequently, an experimentally measured persistence phenotype cannot yet be translated directly into a therapeutic recommendation for an individual clinical isolate. For drug development, experimental persistence models nonetheless provide valuable platforms for identifying compounds and treatment strategies active against antibiotic-surviving populations. Their predictive value, however, depends on the model used: activity against persisters generated or enriched under a single laboratory condition does not necessarily translate to activity against physiologically heterogeneous bacterial populations during infection [117,118]. Candidate anti-persister strategies therefore require validation across complementary systems, from controlled mechanistic models to clinical isolates, biofilms, or host-cell models, and, where appropriate, animal infection models.

## 11. Conclusions and Future Perspectives

Bacterial persistence is often discussed as though it were a discrete cellular phenotype that can be directly observed and quantified. As highlighted throughout this Review, persistence is better understood as a dynamic process extending from the physiological state preceding antibiotic exposure, through survival during treatment and recovery after antibiotic removal, to clonal expansion. No single experimental approach captures this entire continuum in its full biological complexity. Population-based methods provide average measurements that obscure cellular heterogeneity, whereas single-cell approaches reveal individual cellular behavior but typically capture only a limited number of persisters. Enrichment and selection strategies increase experimental accessibility to these rare cells but introduce additional biological assumptions about which cells are likely to become persisters. Each approach therefore provides a different and necessarily partial perspective on persistence.

The evolution of persistence research illustrates how methodological advances have progressively reshaped its biological interpretation. Persistence was initially defined operationally by antibiotic survival and regrowth, whereas subsequent population- and single-cell approaches revealed increasingly diverse physiological states and cellular histories underlying this phenotype. This methodological progression has contributed to the shift from viewing persisters as a uniform dormant population to recognizing persistence as a heterogeneous and dynamic process.

The central message of this Review is that experimental observations cannot be interpreted independently of the methodological and biological framework in which they were obtained. Apparently conflicting conclusions may therefore reflect differences in experimental design and biological context rather than fundamentally different persistence mechanisms.

Combining methods that interrogate complementary stages of the persistence continuum can strengthen evidence for persister identification and mechanistic interpretation. CFU-based measurements and killing curves establish persistence operationally at the population level [1], whereas their combination with time-lapse microscopy can directly relate antibiotic survival to the recovery of individual cells. Similarly, longitudinal single-cell tracking combined with fluorescent reporters or biosensors can associate cellular physiology with subsequent persistence outcomes.

A major methodological challenge is to bridge the molecular depth of omics analyses with the temporal resolution of single-cell approaches. Recent advances in bacterial single-cell transcriptomics provide new opportunities to resolve transcriptional heterogeneity that is obscured by population-level measurements [119,120,121], while proteomic and metabolomic analyses of individual microbial cells are becoming technically accessible [122]. Integrating such molecular analyses with lineage tracking, fluorescent biosensors, or other approaches that provide information about cellular history could help relate molecular states to subsequent survival, recovery, and proliferative fate. However, the rarity of persisters and the limited molecular material available from individual bacterial cells remain important technical challenges [122]. Moreover, molecular characterization that requires cell disruption inherently prevents subsequent observation of the same cell during recovery.

Progress in persistence research will depend not on a single experimental breakthrough, but on integrating complementary methodologies with biological context across the persistence continuum.

## Figures and Tables

**Table 1:** Terminology adopted throughout this Review. Cell designation reflects the level of experimental evidence available at each stage of the persistence continuum rather than fixed physiological states.

Cell Designation	Stage of the Persistence Continuum	Definition
Potential persister cell	Before antibiotic exposure	A bacterial cell before antibiotic exposure that may subsequently prove to be a persister. At this stage, however, no experimental approach can determine whether the cell will survive antibiotic treatment, recover, and resume proliferation.
Surviving cell (survivors)	During antibiotic treatment	A cell that remains intact or detectable after antibiotic exposure according to the experimental assay used. Survival alone does not demonstrate the ability to recover or resume growth.
Recovering cell	After antibiotic removal	A surviving cell that has resumed physiological activity and growth following antibiotic removal. Recovery alone does not necessarily demonstrate long-term proliferative capacity.
Persister	Clonal expansion	A surviving cell that has recovered, resumed proliferation, formed a clonal lineage, and retained antibiotic susceptibility. Within the framework adopted in this Review, only completion of the entire persistence continuum provides experimental confirmation of the persister phenotype.
VBNC (viable but non-culturable cells)	After antibiotic removal/recovery	A viable bacterial cell that remains non-culturable under the conditions used for recovery and therefore does not progress to clonal expansion. Unlike a persister cell, a VBNC cell does not form a colony under these conditions.

**Table 3:** Representative sorting and selection strategies for candidate persister cells based on different physiological characteristics.

Candidate Persister Feature	Sorting/Selection Strategy	Key Studies	Representative Applications	Major Limitation
Antibiotic survivors	β-lactam treatment followed by centrifugation or filtration	[19,38]	Transcriptomic profiling; physiological characterization	Mixed population of persister and VBNC cells
Non-filamenting cells	Cephalexin-induced filamentation followed by filtration	[7]	Awakening dynamics; post-antibiotic recovery	Non-filamenting cells are not exclusively persisters
Non-dividing cells	FACS isolation of highly fluorescent cells following fluorescence dilution (stable GFP/mCherry)	[16,92,93,94]	Awakening dynamics; growth heterogeneity; persister vs. VBNC distinction; origins of persisters from dividing and non-dividing cells	Persisters arise from both non-dividing and dividing cells
Low metabolic activity	FACS isolation of low-fluorescent cells using an unstable GFP reporter under the control of the *rrnB* P1 promoter	[36,41]	ATP homeostasis; metabolism–persistence relationship; gene expression profiling	Low metabolic activity is not unique to persisters
SOS response	FACS isolation of SOS reporter-positive cells	[95]	DNA repair; post-fluoroquinolone recovery dynamics	Reflects recovery capacity rather than persistence itself

FACS—fluorescence-activated cell sorting; GFP—green fluorescent protein.

**Table 4:** Experimental factors determining which aspects of bacterial persistence become accessible for investigation.

Biological or Methodological Filter	Which Bacterial Cells Become Accessible for Analysis?	What Information Can Be Obtained?	What Biological Conclusion Can Be Drawn?
Biological context (*strain background, culture* *organization, nutrient and* *oxygen availability,* *host-associated environment*)	Cells developing under different physiological and ecological conditions	Context-dependent survival and physiological responses	The observed persistence phenotype depends on the biological context and cannot be interpreted as a fixed cellular property.
Population and treatment conditions *(growth phase, inoculum age, cell density, antibiotic class, concentration, exposure time)*	Populations with different physiological histories and probabilities of survival	Initial physiological state, antibiotic survival, and apparent persister frequency	Population state and treatment conditions determine which cells survive antibiotic exposure and are subsequently identified as persisters
Enrichment strategies (*rifampicin pretreatment, hip mutants, nutrient shift,* *antibiotic enrichment*)	Persister-enriched but still heterogeneous populations	Population-level characteristics of cells with increased survival probability	Enrichment increases the probability of detecting persisters but does not isolate a pure persister population.
Cell selection strategies (*non-dividing cells,* *ATP-low cells, intact cells,* *morphology-based sorting*)	Candidate persister cells selected according to predefined physiological criteria	Single-cell characteristics of selected candidate persisters	The analyzed cells reflect the biological assumptions underlying the selection strategy rather than the entire persister population.
Analytical methodology			
*CFU and killing* *assays*	Antibiotic survivors capable of regrowth	Survival frequency	Operational definition of persistence based on antibiotic survival and regrowth.
*Population-based* *analyses*	Bulk bacterial populations or persister- enriched fractions	Average transcriptomic, proteomic, metabolomic, and physiological characteristics	Population averages cannot resolve heterogeneity between persisters and non-persisters.
*Fluorescence* *microscopy*	Individual bacterial cells	Morphology, growth, division, awakening dynamics, lineage, and activity of specific physiological processes	Single-cell analyses reveal physiological heterogeneity and can associate specific cellular states with persistence and awakening.
*Microfluidics and* *lineage tracking*	Individual cells observed throughout the persistence continuum	Cellular history before treatment, survival, awakening, lineage relationships	Cellular history can be directly linked to persistence outcome, demonstrating that persistence develops over time rather than representing a static cellular state.

## Data Availability

No new data were created or analyzed in this study. Data sharing is not applicable to this article.

## References

[1] Balaban N.Q., Helaine S., Lewis K., Ackermann M., Aldridge B., Andersson D.I., Brynildsen M.P., Bumann D., Camilli A., Collins J.J. (2019). Definitions and Guidelines for Research on Antibiotic Persistence. Nat. Rev. Microbiol..

[2] Kaldalu N., Hauryliuk V., Turnbull K.J., La Mensa A., Putrinš M., Tenson T. (2020). *In Vitro* Studies of Persister Cells. Microbiol. Mol. Biol. Rev..

[3] Stojowska-Swędrzyńska K., Laskowska E., Kuczyńska-Wiśnik D. (2026). Molecular Basis of Persister Awakening and Lag-Phase Recovery in *Escherichia coli* After Antibiotic Exposure. Int. J. Mol. Sci..

[4] Umetani M., Fujisawa M., Okura R., Nozoe T., Suenaga S., Nakaoka H., Kussell E., Wakamoto Y. (2025). Observation of Persister Cell Histories Reveals Diverse Modes of Survival in Antibiotic Persistence. elife.

[5] Goormaghtigh F., Van Melderen L. (2019). Single-Cell Imaging and Characterization of *Escherichia coli* Persister Cells to Ofloxacin in Exponential Cultures. Sci. Adv..

[6] Manuse S., Shan Y., Canas-Duarte S.J., Bakshi S., Sun W.S., Mori H., Paulsson J., Lewis K. (2021). Bacterial Persisters Are a Stochastically Formed Subpopulation of Low-Energy Cells. PLoS Biol..

[7] Windels E.M., Ben Meriem Z., Zahir T., Verstrepen K.J., Hersen P., Van den Bergh B., Michiels J. (2019). Enrichment of Persisters Enabled by a Beta-Lactam-Induced Filamentation Method Reveals Their Stochastic Single-Cell Awakening. Commun. Biol..

[8] Wong F., Wilson S., Helbig R., Hegde S., Aftenieva O., Zheng H., Liu C., Pilizota T., Garner E.C., Amir A. (2021). Understanding Beta-Lactam-Induced Lysis at the Single-Cell Level. Front. Microbiol..

[9] Kim K., Wang T., Ma H.R., Şimşek E., Li B., Andreani V., You L. (2023). Mapping Single-cell Responses to Population-level Dynamics during Antibiotic Treatment. Mol. Syst. Biol..

[10] Dörr T., Lewis K., Vulić M. (2009). SOS Response Induces Persistence to Fluoroquinolones in *Escherichia coli*. PLoS Genet..

[11] Kim J., Yamasaki R., Song S., Zhang W., Wood T.K. (2018). Single Cell Observations Show Persister Cells Wake Based on Ribosome Content. Environ. Microbiol..

[12] Bollen C., Dewachter L., Michiels J. (2021). Protein Aggregation as a Bacterial Strategy to Survive Antibiotic Treatment. Front. Mol. Biosci..

[13] Murawski A.M., Brynildsen M.P. (2021). Ploidy Is an Important Determinant of Fluoroquinolone Persister Survival. Curr. Biol..

[14] Lewis K. (2010). Persister Cells. Annu. Rev. Microbiol..

[15] Wood T.K., Knabel S.J., Kwan B.W. (2013). Bacterial Persister Cell Formation and Dormancy. Appl. Environ. Microbiol..

[16] Orman M.A., Brynildsen M.P. (2013). Dormancy Is Not Necessary or Sufficient for Bacterial Persistence. Antimicrob. Agents Chemother..

[17] Bigger J.W. (1944). The Bactericidal Action of Penicillin on *Staphylococcus pyogenes*. Ir. J. Med. Sci..

[18] Balaban N.Q., Merrin J., Chait R., Kowalik L., Leibler S. (2004). Bacterial Persistence as a Phenotypic Switch. Science.

[19] Keren I., Kaldalu N., Spoering A., Wang Y., Lewis K. (2004). Persister Cells and Tolerance to Antimicrobials. FEMS Microbiol. Lett..

[20] Lewis K. (2007). Persister Cells, Dormancy and Infectious Disease. Nat. Rev. Microbiol..

[21] Wang R., Lv J., Chen L., Zhao Y., Du H. (2025). Antifungal Persistence: Clinical Relevance and Mechanisms. PLoS Pathog..

[22] Wang Y., Ma X., Zhao L., He Y., Yu W., Fu S., Ni W., Gao Z. (2022). Heteroresistance Is Associated With in Vitro Regrowth During Colistin Treatment in Carbapenem-Resistant Klebsiella Pneumoniae. Front. Microbiol..

[23] Martinecz A., Clarelli F., Abel S., Abel zur Wiesch P. (2019). Reaction Kinetic Models of Antibiotic Heteroresistance. Int. J. Mol. Sci..

[24] Carvalho G., Guilhen C., Balestrino D., Forestier C., Mathias J. (2017). Relating Switching Rates between Normal and Persister Cells to Substrate and Antibiotic Concentrations: A Mathematical Modelling Approach Supported by Experiments. Microb. Biotechnol..

[25] Stojowska-Swędrzyńska K., Łupkowska A., Kuczyńska-Wiśnik D., Laskowska E. (2021). Antibiotic Heteroresistance in Klebsiella Pneumoniae. Int. J. Mol. Sci..

[26] Wiuff C., Zappala R.M., Regoes R.R., Garner K.N., Baquero F., Levin B.R. (2005). Phenotypic Tolerance: Antibiotic Enrichment of Noninherited Resistance in Bacterial Populations. Antimicrob. Agents Chemother..

[27] Levin-Reisman I., Gefen O., Fridman O., Ronin I., Shwa D., Sheftel H., Balaban N.Q. (2010). Automated Imaging with ScanLag Reveals Previously Undetectable Bacterial Growth Phenotypes. Nat. Methods.

[28] Levin-Reisman I., Balaban N.Q., Michiels J., Fauvart M. (2016). Quantitative Measurements of Type I and Type II Persisters Using ScanLag. Bacterial Persistence: Methods and Protocols.

[29] Proctor R.A., Kriegeskorte A., Kahl B.C., Becker K., Löffler B., Peters G. (2014). *Staphylococcus aureus* Small Colony Variants (SCVs): A Road Map for the Metabolic Pathways Involved in Persistent Infections. Front. Cell. Infect. Microbiol..

[30] Proctor R.A., von Eiff C., Kahl B.C., Becker K., McNamara P., Herrmann M., Peters G. (2006). Small Colony Variants: A Pathogenic Form of Bacteria That Facilitates Persistent and Recurrent Infections. Nat. Rev. Microbiol..

[31] Fisher R.A., Gollan B., Helaine S. (2017). Persistent Bacterial Infections and Persister Cells. Nat. Rev. Microbiol..

[32] Welch D.F., Guruswamy A.P., Sides S.J., Shaw C.H., Gilchrist M.J. (1993). Timely Culture for Mycobacteria Which Utilizes a Microcolony Method. J. Clin. Microbiol..

[33] Dhillon J., Fourie P.B., Mitchison D.A. (2014). Persister Populations of Mycobacterium Tuberculosis in Sputum That Grow in Liquid but Not on Solid Culture Media. J. Antimicrob. Chemother..

[34] Van den Bergh B., Fauvart M., Michiels J. (2017). Formation, Physiology, Ecology, Evolution and Clinical Importance of Bacterial Persisters. FEMS Microbiol. Rev..

[35] de Knegt G.J., Dickinson L., Pertinez H., Evangelopoulos D., McHugh T.D., Bakker-Woudenberg I.A.J.M., Davies G.R., de Steenwinkel J.E.M. (2017). Assessment of Treatment Response by Colony Forming Units, Time to Culture Positivity and the Molecular Bacterial Load Assay Compared in a Mouse Tuberculosis Model. Tuberculosis.

[36] Shah D., Zhang Z., Khodursky A., Kaldalu N., Kurg K., Lewis K. (2006). Persisters: A Distinct Physiological State of *E. coli*. BMC Microbiol..

[37] Matsumoto S., Kawai Y., Miyagawa S., Iwamoto Y., Okuda S., Sánchez-Gorostiaga A., Vicente M., Tsuneda S. (2018). Unique Transcriptional Profile of Native Persisters in *Escherichia coli*. J. Biosci. Bioeng..

[38] Rahman K.M.T., Amaratunga R., Butzin X.Y., Singh A., Hossain T., Butzin N.C. (2025). Rethinking Dormancy: Antibiotic Persisters Are Metabolically Active, Non-Growing Cells. Int. J. Antimicrob. Agents.

[39] Maisonneuve E., Gerdes K. (2014). Molecular Mechanisms Underlying Bacterial Persisters. Cell.

[40] Germain E., Castro-Roa D., Zenkin N., Gerdes K. (2013). Molecular Mechanism of Bacterial Persistence by HipA. Mol. Cell.

[41] Shan Y., Brown Gandt A., Rowe S.E., Deisinger J.P., Conlon B.P., Lewis K. (2017). ATP-Dependent Persister Formation in *Escherichia coli*. mBio.

[42] Mok W.W.K., Park J.O., Rabinowitz J.D., Brynildsen M.P. (2015). RNA Futile Cycling in Model Persisters Derived from MazF Accumulation. mBio.

[43] Orman M.A., Brynildsen M.P. (2015). Inhibition of Stationary Phase Respiration Impairs Persister Formation in *E. coli*. Nat. Commun..

[44] Svenningsen M.S., Veress A., Harms A., Mitarai N., Semsey S. (2019). Birth and Resuscitation of (p)PpGpp Induced Antibiotic Tolerant Persister Cells. Sci. Rep..

[45] Liu S., Wu N., Zhang S., Yuan Y., Zhang W., Zhang Y. (2017). Variable Persister Gene Interactions with (p)PpGpp for Persister Formation in *Escherichia coli*. Front. Microbiol..

[46] Sivapragasam S. (2025). The Emerging Role of (p)PpGpp in DNA Repair and Associated Bacterial Survival against Fluoroquinolones. Gene Expr..

[47] Leszczynska D., Matuszewska E., Kuczynska-Wisnik D., Furmanek-Blaszk B., Laskowska E. (2013). The Formation of Persister Cells in Stationary-Phase Cultures of *Escherichia coli* Is Associated with the Aggregation of Endogenous Proteins. PLoS ONE.

[48] Yu J., Liu Y., Yin H., Chang Z. (2019). Regrowth-Delay Body as a Bacterial Subcellular Structure Marking Multidrug-Tolerant Persisters. Cell Discov..

[49] Bollen C., Louwagie S., Deroover F., Duverger W., Khodaparast L., Khodaparast L., Hofkens D., Schymkowitz J., Rousseau F., Dewachter L. (2025). Composition and Liquid-to-Solid Maturation of Protein Aggregates Contribute to Bacterial Dormancy Development and Recovery. Nat. Commun..

[50] Dewachter L., Bollen C., Wilmaerts D., Louwagie E., Herpels P., Matthay P., Khodaparast L., Khodaparast L., Rousseau F., Schymkowitz J. (2021). The Dynamic Transition of Persistence toward the Viable but Nonculturable State during Stationary Phase Is Driven by Protein Aggregation. mBio.

[51] Sulaiman J.E., Lam H. (2020). Proteomic Investigation of Tolerant *Escherichia coli* Populations from Cyclic Antibiotic Treatment. J. Proteome Res..

[52] Sulaiman J.E., Hao C., Lam H. (2018). Specific Enrichment and Proteomics Analysis of *Escherichia coli* Persisters from Rifampin Pretreatment. J. Proteome Res..

[53] Sulaiman J.E., Lam H. (2019). Application of Proteomics in Studying Bacterial Persistence. Expert Rev. Proteom..

[54] Semanjski M., Gratani F.L., Englert T., Nashier P., Beke V., Nalpas N., Germain E., George S., Wolz C., Gerdes K. (2021). Proteome Dynamics during Antibiotic Persistence and Resuscitation. mSystems.

[55] Urbaniec J., Xu Y., Hu Y., Hingley-Wilson S., McFadden J. (2022). Phenotypic Heterogeneity in Persisters: A Novel “hunker” Theory of Persistence. FEMS Microbiol. Rev..

[56] Dawson E., Şimşek E., Kim M., Kim M. (2021). Observing Bacterial Persistence at Single-Cell Resolution. Bacterial Persistence.

[57] Fang X., Allison K.R. (2023). Resuscitation Dynamics Reveal Persister Partitioning after Antibiotic Treatment. Mol. Syst. Biol..

[58] Yamasaki R., Song S., Benedik M.J., Wood T.K. (2020). Persister Cells Resuscitate Using Membrane Sensors That Activate Chemotaxis, Lower CAMP Levels, and Revive Ribosomes. iScience.

[59] Priest D.G., Tanaka N., Tanaka Y., Taniguchi Y. (2017). Micro-Patterned Agarose Gel Devices for Single-Cell High-Throughput Microscopy of *E. coli* Cells. Sci. Rep..

[60] Moffitt J.R., Lee J.B., Cluzel P. (2012). The Single-Cell Chemostat: An Agarose-Based, Microfluidic Device for High-Throughput, Single-Cell Studies of Bacteria and Bacterial Communities. Lab Chip.

[61] Wang P., Robert L., Pelletier J., Dang W.L., Taddei F., Wright A., Jun S. (2010). Robust Growth of *Escherichia coli*. Curr. Biol..

[62] Bamford R.A., Smith A., Metz J., Glover G., Titball R.W., Pagliara S. (2017). Investigating the Physiology of Viable but Non-Culturable Bacteria by Microfluidics and Time-Lapse Microscopy. BMC Biol..

[63] Le Quellec L., Aristov A., Gutiérrez Ramos S., Amselem G., Bos J., Baharoglu Z., Mazel D., Baroud C.N. (2024). Measuring Single-Cell Susceptibility to Antibiotics within Monoclonal Bacterial Populations. PLoS ONE.

[64] Oms T., Schlechtweg T., Cayron J., Van Melderen L. (2023). Population and Single-Cell Analysis of Antibiotic Persistence in *Escherichia coli*. J. Vis. Exp..

[65] Barrett T.C., Mok W.W.K., Murawski A.M., Brynildsen M.P. (2019). Enhanced Antibiotic Resistance Development from Fluoroquinolone Persisters after a Single Exposure to Antibiotic. Nat. Commun..

[66] Goode O., Smith A., Łapińska U., Bamford R., Kahveci Z., Glover G., Attrill E., Carr A., Metz J., Pagliara S. (2021). Heterologous Protein Expression Favors the Formation of Protein Aggregates in Persister and Viable but Nonculturable Bacteria. ACS Infect. Dis..

[67] Miyaue S., Suzuki E., Komiyama Y., Kondo Y., Morikawa M., Maeda S. (2018). Bacterial Memory of Persisters: Bacterial Persister Cells Can Retain Their Phenotype for Days or Weeks After Withdrawal From Colony–Biofilm Culture. Front. Microbiol..

[68] Vedelaar S.R., Radzikowski J.L., Heinemann M., Kim M. (2021). A Robust Method for Generating, Quantifying, and Testing Large Numbers of *Escherichia coli* Persisters. Bacterial Persistence.

[69] Moyed H.S., Bertrand K.P. (1983). HipA, a Newly Recognized Gene of *Escherichia coli* K-12 That Affects Frequency of Persistence after Inhibition of Murein Synthesis. J. Bacteriol..

[70] Kwan B.W., Valenta J.A., Benedik M.J., Wood T.K. (2013). Arrested Protein Synthesis Increases Persister-like Cell Formation. Antimicrob. Agents Chemother..

[71] Grassi L., Di Luca M., Maisetta G., Rinaldi A.C., Esin S., Trampuz A., Batoni G. (2017). Generation of Persister Cells of Pseudomonas Aeruginosa and *Staphylococcus aureus* by Chemical Treatment and Evaluation of Their Susceptibility to Membrane-Targeting Agents. Front. Microbiol..

[72] Narayanaswamy V.P., Keagy L.L., Duris K., Wiesmann W., Loughran A.J., Townsend S.M., Baker S. (2018). Novel Glycopolymer Eradicates Antibiotic- and CCCP-Induced Persister Cells in Pseudomonas Aeruginosa. Front. Microbiol..

[73] Petersen M.E., Hansen L.K., Mitkin A.A., Kelly N.M., Wood T.K., Jørgensen N.P., Østergaard L.J., Meyer R.L. (2024). A High-Throughput Assay Identifies Molecules with Antimicrobial Activity against Persister Cells. J. Med. Microbiol..

[74] Aedo S.J., Orman M.A., Brynildsen M.P. (2019). Stationary Phase Persister Formation in *Escherichia coli* Can Be Suppressed by Piperacillin and PBP3 Inhibition. BMC Microbiol..

[75] Ikeda H., Maeda S. (2024). Characterization of *Escherichia coli* Persisters from Biofilm Culture: Multiple Dormancy Levels and Multigenerational Memory in Formation. Microorganisms.

[76] Amato S.M., Brynildsen M.P. (2014). Nutrient Transitions Are a Source of Persisters in *Escherichia coli* Biofilms. PLoS ONE.

[77] Radzikowski J.L., Vedelaar S., Siegel D., Ortega Á.D., Schmidt A., Heinemann M. (2016). Bacterial Persistence Is an Active ΣS Stress Response to Metabolic Flux Limitation. Mol. Syst. Biol..

[78] Amato S.M., Orman M.A., Brynildsen M.P. (2013). Metabolic Control of Persister Formation in *Escherichia coli*. Mol. Cell.

[79] Cho H., Uehara T., Bernhardt T.G. (2014). Beta-Lactam Antibiotics Induce a Lethal Malfunctioning of the Bacterial Cell Wall Synthesis Machinery. Cell.

[80] Blattman S.B., Jiang W., McGarrigle E.R., Liu M., Oikonomou P., Tavazoie S. (2024). Identification and Genetic Dissection of Convergent Persister Cell States. Nature.

[81] Ye Y., Tian Y., Duan M., Zhu W., Wu J., Chen Y., Xu F., Zhao X., Drlica K., Hong Y. (2025). Antibiotic-Persistent Bacterial Cells Exhibiting Low-Level ROS Are Eradicated by ROS-Independent Membrane Disruption. mBio.

[82] Cho J., Rogers J., Kearns M., Leslie M., Hartson S.D., Wilson K.S. (2015). *Escherichia coli* Persister Cells Suppress Translation by Selectively Disassembling and Degrading Their Ribosomes. Mol. Microbiol..

[83] Kim J., Chowdhury N., Yamasaki R., Wood T.K. (2018). Viable but Non-culturable and Persistence Describe the Same Bacterial Stress State. Environ. Microbiol..

[84] Kim H., Song S. (2026). Ribosome State Distributions Define *Escherichia coli* Persister Physiology: Links to Formation, Stress Responses, and Resuscitation Dynamics. Microb. Biotechnol..

[85] Cui P., Niu H., Shi W., Zhang S., Zhang W., Zhang Y. (2018). Identification of Genes Involved in Bacteriostatic Antibiotic-Induced Persister Formation. Front. Microbiol..

[86] Fernández-García L., Kirigo J., Huelgas-Méndez D., Benedik M.J., Tomás M., García-Contreras R., Wood T.K. (2024). Phages Produce Persisters. Microb. Biotechnol..

[87] Song S., Wood T.K. (2020). PpGpp Ribosome Dimerization Model for Bacterial Persister Formation and Resuscitation. Biochem. Biophys. Res. Commun..

[88] Song S., Wood T.K. (2020). Persister Cells Resuscitate via Ribosome Modification by 23S RRNA Pseudouridine Synthase RluD. Environ. Microbiol..

[89] Park G., Kim H., Song S. (2026). A Novel Small Molecule Accelerates Early Persister Regrowth and Potentiates Antibiotic Killing via MdtL–DcrB. Microb. Biotechnol..

[90] Kwan B.W., Chowdhury N., Wood T.K. (2015). Combatting Bacterial Infections by Killing Persister Cells with Mitomycin C. Environ. Microbiol..

[91] Kawano A., Yamasaki R., Sakakura T., Takatsuji Y., Haruyama T., Yoshioka Y., Ariyoshi W. (2020). Reactive Oxygen Species Penetrate Persister Cell Membranes of *Escherichia coli* for Effective Cell Killing. Front. Cell. Infect. Microbiol..

[92] Roostalu J., Jõers A., Luidalepp H., Kaldalu N., Tenson T. (2008). Cell Division in *Escherichia coli* Cultures Monitored at Single Cell Resolution. BMC Microbiol..

[93] Orman M.A., Brynildsen M.P. (2013). Establishment of a Method To Rapidly Assay Bacterial Persister Metabolism. Antimicrob. Agents Chemother..

[94] Mohiuddin S.G., Kavousi P., Orman M.A. (2020). Flow-Cytometry Analysis Reveals Persister Resuscitation Characteristics. BMC Microbiol..

[95] Völzing K.G., Brynildsen M.P. (2015). Stationary-Phase Persisters to Ofloxacin Sustain DNA Damage and Require Repair Systems Only during Recovery. mBio.

[96] Luidalepp H., Jõers A., Kaldalu N., Tenson T. (2011). Age of Inoculum Strongly Influences Persister Frequency and Can Mask Effects of Mutations Implicated in Altered Persistence. J. Bacteriol..

[97] Jõers A., Kaldalu N., Tenson T. (2010). The Frequency of Persisters in *Escherichia coli* Reflects the Kinetics of Awakening from Dormancy. J. Bacteriol..

[98] Udekwu K.I., Parrish N., Ankomah P., Baquero F., Levin B.R. (2009). Functional Relationship between Bacterial Cell Density and the Efficacy of Antibiotics. J. Antimicrob. Chemother..

[99] Michiels J.E., Van den Bergh B., Verstraeten N., Michiels J. (2016). Molecular Mechanisms and Clinical Implications of Bacterial Persistence. Drug Resist. Updates.

[100] Fauvart M., de Groote V.N., Michiels J. (2011). Role of Persister Cells in Chronic Infections: Clinical Relevance and Perspectives on Anti-Persister Therapies. J. Med. Microbiol..

[101] Lebeaux D., Ghigo J.-M., Beloin C. (2014). Biofilm-Related Infections: Bridging the Gap between Clinical Management and Fundamental Aspects of Recalcitrance toward Antibiotics. Microbiol. Mol. Biol. Rev..

[102] Alexandersen N.R., Nielsen K.L., Häussler S., Bjarnsholt T., Schønning K. (2025). Antibiotic Tolerance and Persistence in Clinical Isolates of *Escherichia coli* Evaluated by High-Resolution Time-Kill Assays. Microbiol. Spectr..

[103] Barth V.C., Rodrigues B.Á., Bonatto G.D., Gallo S.W., Pagnussatti V.E., Ferreira C.A.S., de Oliveira S.D. (2013). Heterogeneous Persister Cells Formation in Acinetobacter Baumannii. PLoS ONE.

[104] Mulcahy L.R., Burns J.L., Lory S., Lewis K. (2010). Emergence of Pseudomonas Aeruginosa Strains Producing High Levels of Persister Cells in Patients with Cystic Fibrosis. J. Bacteriol..

[105] Huemer M., Mairpady Shambat S., Bergada-Pijuan J., Söderholm S., Boumasmoud M., Vulin C., Gómez-Mejia A., Antelo Varela M., Tripathi V., Götschi S. (2021). Molecular Reprogramming and Phenotype Switching in *Staphylococcus aureus* Lead to High Antibiotic Persistence and Affect Therapy Success. Proc. Natl. Acad. Sci. USA.

[106] Li P., Yao S., Dincer S., Ozdenefe M.S., Takci H.A.M. (2025). Persisters of Bacterial Biofilms. Exploring Bacterial Biofilms.

[107] Stewart P.S., Franklin M.J. (2008). Physiological Heterogeneity in Biofilms. Nat. Rev. Microbiol..

[108] Spoering A.L., Lewis K. (2001). Biofilms and Planktonic Cells of *Pseudomonas Aeruginosa* Have Similar Resistance to Killing by Antimicrobials. J. Bacteriol..

[109] Helaine S., Cheverton A.M., Watson K.G., Faure L.M., Matthews S.A., Holden D.W. (2014). Internalization of *Salmonella* by Macrophages Induces Formation of Nonreplicating Persisters. Science.

[110] Stapels D.A.C., Hill P.W.S., Westermann A.J., Fisher R.A., Thurston T.L., Saliba A.-E., Blommestein I., Vogel J., Helaine S. (2018). Salmonella Persisters Undermine Host Immune Defenses during Antibiotic Treatment. Science.

[111] Fanous J., Claudi B., Tripathi V., Li J., Goormaghtigh F., Bumann D. (2025). Limited Impact of Salmonella Stress and Persisters on Antibiotic Clearance. Nature.

[112] Gollan B., Grabe G., Michaux C., Helaine S. (2019). Bacterial Persisters and Infection: Past, Present, and Progressing. Annu. Rev. Microbiol..

[113] Fisher R.A., Cheverton A.M., Helaine S., Michiels J., Fauvart M. (2016). Analysis of Macrophage-Induced Salmonella Persisters. Bacterial Persistence: Methods and Protocols.

[114] Michaux C., Ronneau S., Helaine S., Verstraeten N., Michiels J. (2021). Studying Antibiotic Persistence During Infection. Bacterial Persistence: Methods and Protocols.

[115] Kuehl R., Morata L., Meylan S., Mensa J., Soriano A. (2020). When Antibiotics Fail: A Clinical and Microbiological Perspective on Antibiotic Tolerance and Persistence of Staphylococcus Aureus. J. Antimicrob. Chemother..

[116] Deventer A.T., Stevens C.E., Stewart A., Hobbs J.K. (2024). Antibiotic Tolerance among Clinical Isolates: Mechanisms, Detection, Prevalence, and Significance. Clin. Microbiol. Rev..

[117] Defraine V., Fauvart M., Michiels J. (2018). Fighting Bacterial Persistence: Current and Emerging Anti-Persister Strategies and Therapeutics. Drug Resist. Updates.

[118] Niu H., Gu J., Zhang Y. (2024). Bacterial Persisters: Molecular Mechanisms and Therapeutic Development. Signal Transduct. Target. Ther..

[119] Imdahl F., Vafadarnejad E., Homberger C., Saliba A.-E., Vogel J. (2020). Single-Cell RNA-Sequencing Reports Growth-Condition-Specific Global Transcriptomes of Individual Bacteria. Nat. Microbiol..

[120] Blattman S.B., Jiang W., Oikonomou P., Tavazoie S. (2020). Prokaryotic Single-Cell RNA Sequencing by in Situ Combinatorial Indexing. Nat. Microbiol..

[121] Kuchina A., Brettner L.M., Paleologu L., Roco C.M., Rosenberg A.B., Carignano A., Kibler R., Hirano M., DePaolo R.W., Seelig G. (2021). Microbial Single-Cell RNA Sequencing by Split-Pool Barcoding. Science.

[122] Schirmer M., Dusny C. (2023). Microbial Single-Cell Mass Spectrometry: Status, Challenges, and Prospects. Curr. Opin. Biotechnol..

